# Efficacy and safety of switching from branded to generic antiretrovirals in virologically suppressed HIV-infected patients

**DOI:** 10.1371/journal.pone.0182007

**Published:** 2017-08-01

**Authors:** Nicola Gianotti, Andrea Poli, Laura Galli, Michela Franzin, Patrizia Tadini, Nadia Galizzi, Alessia Carbone, Marco Merli, Camilla Muccini, Chiara Oltolini, Andrea Andolina, Vincenzo Spagnuolo, Adriano Lazzarin, Antonella Castagna

**Affiliations:** 1 Infectious Diseases, San Raffaele Scientific Institute, Milano, Italy; 2 Pharmacy, San Raffaele Scientific Institute, Milano, Italy; 3 Università Vita-Salute San Raffaele, Milano, Italy; National and Kapodistrian University of Athens, GREECE

## Abstract

**Background:**

Aim of this study was to evaluate the efficacy and the safety of switching from branded to generic antiretrovirals in patients with HIV-RNA <50 copies/mL.

**Methods:**

Matched-cohort study of patients followed at a single clinical center. Since September 2014, all patients with HIV-RNA <50 copies/mL who were receiving branded lamivudine or zidovudine/lamivudine or efavirenz were switched to the generic compound (switchers) and matched, in a ratio 1:1, for age (±5 years), gender, anti-HCV antibodies, nadir and (±50 cells/μL) baseline CD4+ count (±100 cells/μL), duration of antiretroviral therapy (±1 year), with patients with HIV-RNA <50 copies/mL, on treatment with unavailable generic compounds (non-switchers). Incidence rates (IR) of different outcomes were calculated and compared by Poisson regression model. A confirmed HIV-RNA ≥50 copies/mL defined virological failure; any change in the antiretroviral regimen was defined as treatment discontinuation.

**Results:**

Four hundred forty patients were switched to generic compounds (268 [61%] on lamivudine, 65 [15%] on zidovudine/lamivudine, 87 [20%] on efavirenz and 20 [4%] on efavirenz and either lamivudine or zidovudine/lamivudine). Over a median follow-up of 15.0 (12.1–15.7) months, virological failure occurred in four switchers (IR: 0.07 [0.02–0.18]/100-person months of follow-up [PMFU]) and in ten non-switchers (IR: 0.20 [0.10–0.35]/100-PMFU) (p = 0.0003), while treatment discontinuation occurred in 118 switchers (IR: 2.05 [1.70–2.44]/100-PMFU) and in 128 non-switchers (IR: 2.37 [1.99–2.81]/100-PMFU) (p = 0.699).

**Conclusions:**

After more than one year of follow-up, we found no evidence of increased risk of reduced efficacy or increased toxicity after switching from branded to generic lamivudine or zidovudine/lamivudine or efavirenz.

## Introduction

Generic anti-retroviral drugs are commonly used and have contributed in a large measure to the scaling up of antiretroviral therapy (ART) in the developing world [1], thus leading to a consistent reduction in mortality for HIV and AIDS in these countries [1–4].

Despite their wide use, the quality and efficacy of generic drugs is questioned because it is based on bioequivalence studies with wide confidence intervals [5–7] rather than on clinical evidence.

Generic drugs have been studied in the treatment of different diseases, including severe infections, with results usually comparable with that of branded ones [8–11]. However, comparative data on generic antiretrovirals are still limited to one study: in HIV-infected adults starting zidovudine-based first-line therapy in Zambia, clinical outcomes, including mortality, hemoglobin and CD4+ cell count changes, did not differ from those observed in patients who received proprietary formulations of the same drugs [12].

A number of other studies have investigated the effectiveness of generic antiretrovirals, but the population treated with these drugs was not compared with a similar population receiving branded formulations [13–20]; furthermore, all of the studies on generic antiretrovirals were performed only in resource-limited countries and data on virological efficacy are scarse.

Finally, also post-approval safety monitoring data are limited [5], particularly outside the setting of resource-limited countries.

Objectives of this study were to evaluate the efficacy and safety of generic antiretrovirals in our clinical center.

## Methods

This was a matched-cohort study of patients followed at a single clinical center.

Since the beginning of September 2014, all HIV-infected patients followed at the San Raffaele Scientific Institute, Milano, who were receiving branded lamivudine or zidovudine/lamivudine or efavirenz were switched to the generic compound. The switch to the generic compounds was based on the modification of the drugs supply by the Hospital Pharmacy and not on clinical reasons.

Data for this study were collected as part of the routine clinical activity; for this reason, there was not a specific approval by an ethical body or review board. Data recorded in the database of the Infectious Diseases Department of the San Raffaele Scientific Institute (IDD-HSR) in Milano, Italy, were used for the analyses. At their first visit in our clinic, subjects provide written informed consent to include their clinical and laboratory data in the IDD-HSR for scientific purposes. Information on prescribed antiretroviral and concomitant drugs (type, dosage, date of start or stop) are prospectively recorded into the database at each visit by the treating physician and then checked by skilled data managers.

All patients switched from branded to generic lamivudine or zidovudine/lamivudine or efavirenz (switchers), with HIV-RNA <50 copies/mL and at least one viral load assessed after switch, were matched (in a 1:1 ratio) to patients (non-switchers) with HIV-RNA <50 copies/mL, on treatment with unavailable generic compounds.

Matching variables were: age (± 5 years), gender, Ab anti-HCV, nadir CD4+ count (± 50 cells/μL), baseline CD4+ count (± 100 cells/μL), index date, duration of antiretroviral therapy (± 1 year). An index date (within the index month) was assigned to each switcher and non-switcher. For switcher, the index date was the date of switch to generic compounds. For non-switchers, the assigned index date allowed matching to their corresponding switchers.

Lost to follow-up was defined as lack of visits or of laboratory test for at least nine months. Follow-up was censored at the outcome date (if occurred) or at the freezing date (February 17^th^, 2016).

Virological outcomes were defined as follows: viral blip = single (not confirmed) HIV-RNA ≥50 copies/mL; virological failure (VF) = confirmed HIV-RNA ≥50 copies/mL; residual viremia = any detectable HIV-RNA below 50 copies/mL, as assed by Abbot Real-Time PCR.

Time spent with residual viremia was calculated as the proportion of time with residual viremia on observed follow-up. If between two observations the viremia changed from undetectable to residual or vice-versa, the time spent considered in this interval was the half. The mathematical formula was:

$$T_{\%}=\frac{\sum_{i=1}^{i=j}\left(\frac{t_{i}-t_{i-1}}{a}\right)}{t_{tot}}\cdot 100$$

where t is the timepoint of the i^th^ observation, ranging from 1 to j (last observation) and t_tot_ is the cumulative patient’s follow-up. If, during the time interval (t_i_-t_i_-1), viremia changed from undetectable to RV or vice-versa then a = 2, else a = 1. Any change in the antiretroviral regimen was defined as treatment discontinuation.

Descriptive data are expressed as median (interquartile range, IQR) of frequency (%), as appropriate.

Chi-square and Mann-Whitney test were used to evaluate differences between the two groups for categorical and continuous variables respectively.

Incidence rates (95% confidence intervals [CI]) per 100 person months of follow-up (PMFU) of different outcomes, according to the use of at least one generic compound, were calculated and compared by univariate Poisson regression.

All of the statistical tests were two-sided at 5% level, and were performed using SAS Software (release 9.2; SAS Institute).

## Results

Four hundred forty patients were switched to generic compounds (268 [61%] switched lamivudine, 65 [15%] switched zidovudine/lamivudine, 87 [20%] switched efavirenz and 20 [4%] switched efavirenz and either lamivudine or zidovudine/lamivudine); their baseline characteristics (along with those of the 440 paired non-switchers) are illustrated in Table 1.

**Table 1:** Baseline characteristics.

	Switchers N = 440	Non-switchers N = 440	p-value
**Age *(years)*, *median (IQR)***	51.9 (47.5–57.9)	51.6 (48.2–57.7)	0.789
**Male gender, *n (%)***	332 (76%)	332 (76%)	-
**Risk factor, *n (%)***			0.522
**Man who have sex with men**	133 (30%)	149 (34%)	
**Heterosexual**	95 (22%)	98 (22%)	
**IDU**	90 (20%)	88 (20%)	
**Other/Unknown**	122 (28%)	105 (24%)	
**Years of since HIV diagnosis, *median (IQR)***	19.5 (12.8–23.9)	19.1 (12.8–24.9)	0.967
**Positive anti-HCV antibodies, *n(%)***	124 (28%)	124 (28%)	-
**AIDS diagnosis, *n (%)***	109 (25%)	91 (21%)	0.171
**Years of ART, *median (IQR)***	15.6 (9.3–18.7)	16.2 (9.8–18.9)	0.765
**Nadir CD4+ count *(cells/μL)*, *median (IQR)***	213 (102–326)	217 (103–326)	0.797
**CD4+ count *(cells/μL)*, *median (IQR)***	709 (547–926)	706 (540–900)	0.583
**Residual viremia, *n (%)***	89 (20%)	106 (24%)	0.194
**Drug switched from branded to generic, *n (%)***			-
**3TC**	268 (61%)		
**3TC/AZT**	65 (15%)		
**EFV**	87 (20%)		
**EFV and 3TC or EFV and 3TC/AZT**	20 (4%)		
**Type of ART regimen according to the drug class, *n (%)***			0.004
**PI-based**	249 (57%)	195 (44%)	
**NNRTI-based**	120 (27%)	151 (34%)	
**InSTI-based**	61 (14%)	83 (19%)	
**NRTI-based**	10 (2%)	11 (3%)	
**Type of ART regimen, *n (%)***			<0.0001
**Dual therapy**	175 (40%)	37 (8%)	
**Three or more drugs (standard ART)**	265 (60%)	403 (92%)	
**Years since the start of the ongoing regimen, *median (IQR)***	3.0 (1.3–6.0)	3.6 (1.5–5.7)	0.229

IDU = intravenous drug user; HCV = Hepatitis C Virus; ART = antiretroviral therapy; 3TC = lamivudine; AZT = zidovudine; EFV = efavirenz; PI = protease inhibitor; PI/r = protease inhibitor boosted with ritonavir; NRTI = nucleoside reverse transcriptase inhibitor; NNRTI = non-nucleoside reverse transcriptase inhibitor; InSTI = integrase strand transfer inhibitor.

Differences in baseline characteristics were observed only with regard to the type of regimen: patients switched to generic compound were more frequently treated with a PI/r-based dual regimens (40% vs. 8%) and less frequently with a triple regimen (60% vs. 92%).

**Fig 1:**
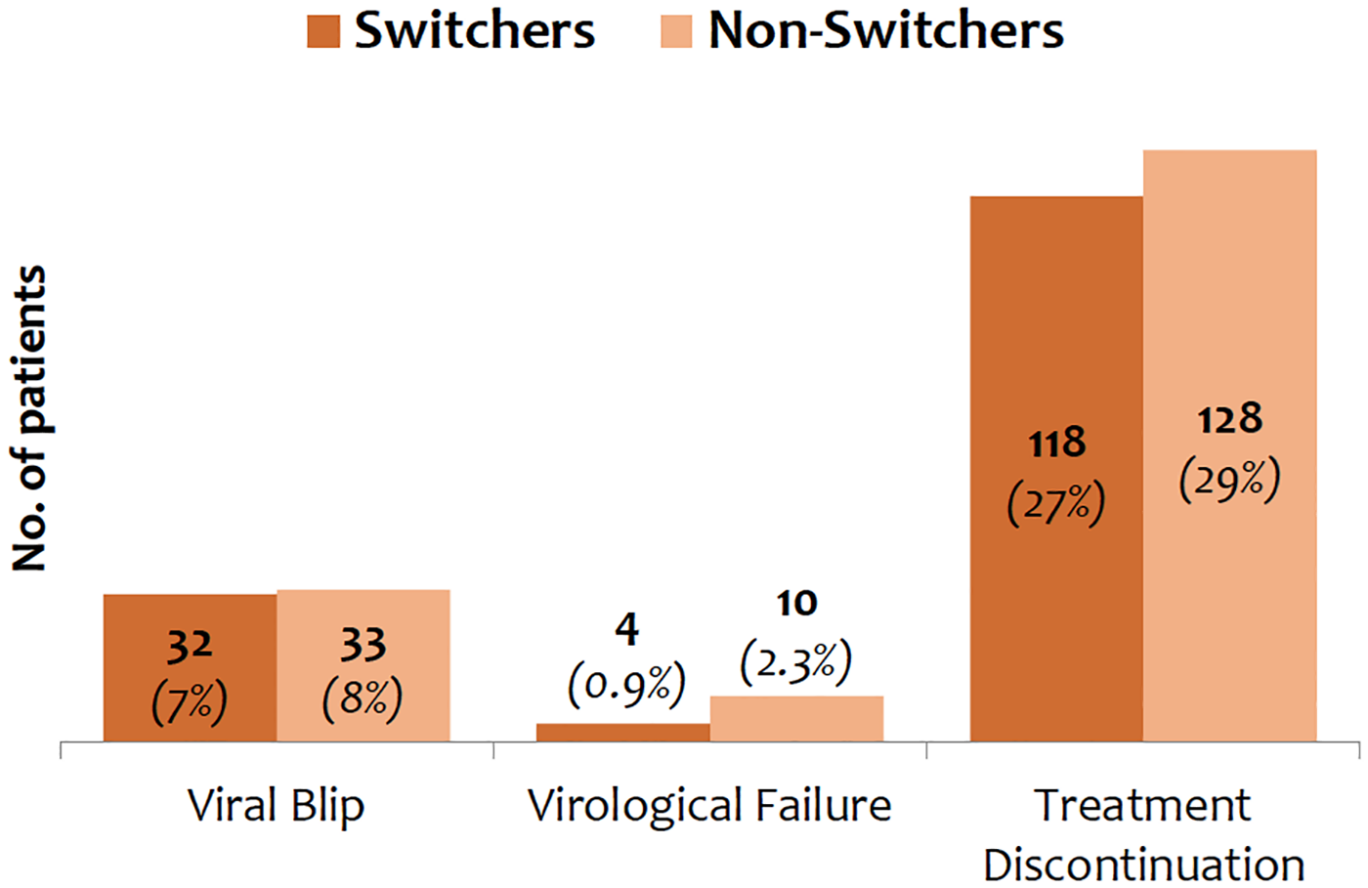
Main outcomes among switchers to generic compounds and non-switchers.

Switchers and non-switchers were followed for a median follow-up of 15.0 (12.2–15.7) and 15.0 (11.8–15.7) months, respectively (p = 0.280). Two (0.1%) and 12 (2.7%) switchers and non-switchers (p = 0.012) were lost to follow-up. VF occurred in 4 (0.9%) switchers and in 10 (2.3%) non-switchers, while treatment discontinuation occurred in 118 (27%) switchers and in 128 (29%) non-switchers. Viral blips occurred in 32 (7%) and 33 (8%) switchers and non-switchers, respectively (Fig 1). The time spent with residual viremia was remarkably similar in the two groups (29% [IQR: 16%-52%] vs. 30% [IQR: 15%-62%]; p = 0.377).

Incidence rates of all outcomes are detailed in Table 2.

**Table 2:** Incidence rates and relative risks of the main outcomes.

	Switchers (N = 440)			Non-switchers (N = 440)			RR *[switchers vs*. *non-switchers (95% CI)]*	p-value
	N	PMFU^a^	IR *(per 100 PMFU*^a^*)*	N	PMFU^a^	IR *(per 100 PMFU*^a^*)*		
**Treatment discontinuation**	118^b^	5769	2.05 (1.70–2.44)	128^b^	5391	2.37 (1.99–2.81)	0.89 (0.49–1.63)	0.699
**Virological failure**	4^b^	5530	0.07 (0.02–0.18)	10^b^	5123	0.20 (0.10–0.35)	0.37 (0. 21–0.66)	0.0003
**Cumulative viral blips**	34^c^	5769	0.59 (0.42–0.81)	37^c^	5391	0.69 (0.49–0.94)	0.93 (0.64–1.36)	0.720

^a)^ Person months of follow-up;

^b)^ No. patients experiencing the event;

^c)^ No. events during follow-up.

Relative risks (95%CI) for switchers vs. non-switchers, of VF, treatment discontinuation and viral blips were 0.37 (0.21–0.66; p = 0.0003), 0.89 (0.49–1.63; p = 0.699) and 0.93 (0.64–1.36; p = 0.720), respectively.

Among switchers, treatment discontinuations occurred in 83 (IR: 2.4 [1.9–2.9]/100-PMFU), 11 (IR: 1.2 [0.6–2.0]/100-PMFU), 19 (IR: 1.7 [1.0–2.6]/100-PMFU), and in five (IR: 1.9 [0.6–3.9]/100-PMFU) patients who switched lamivudine, zidovudine/lamivudine, efavirenz and efavirenz and lamivudine, respectively (p = 0.744). A discontinuation of only the generic drug occurred in nine (IR: 0.3 [0.1–0.5]/100-PMFU) of patients who were receiving lamivudine, in four (IR: 0.4 [0.1–1.0]/100-PMFU) of those who were receiving zidovudine/lamivudine, in eight (IR: 0.7 [0.3–1.3]/100-PMFU) of those who were receiving efavirenz and in one (IR: 0.4 [0.001–1.5]/100-PMFU) of those who were receiving efavirenz and lamivudine (p = 0.763).

Toxicity was the main reason for treatment discontinuation in both groups (31% among switchers and 35% among non-switchers), followed by treatment simplification (25% among switchers and 31% among non-switchers) and drug-drug interaction, which accounted for 14% of treatment discontinuation among switchers and for 8% of treatment discontinuations among non-switchers.

Considering only patients treated with a standard “triple” ART, two and ten VFs occurred among switchers and non-switchers, respectively (IR: 0.06 [0.01–0.20]/100PMFU vs 0.21 [0.11–0.38]/100PMFU; p = 0.002), while treatment discontinuation occurred in 66 (IR: 1.9 [1.5–2.4]/100-PMFU) switchers and in 117 (IR: 2.4 [2.0–2.8]/100-PMFU) non-switchers (p = 0.850).

## Discussion

In this observational study we compared patients switched to generic antiretrovirals with a matched population who continued taking branded drugs and we did not find evidence of reduced efficacy or increased toxicity related to switch from branded to generic antiretroviral drugs.

Indeed, the incidence of VF was lower in patients switched to generic antiretrovirals. We tried to investigate more in depth the virological outcome in the two groups by looking at both the incidence of viral blips and the time spent with residual viremia and we did not find evidence of lower efficacy with generic formulations. Residual viremia was never studied in patients receiving generic antiretrovirals and is of particular interest because its presence has been associated with a higher risk of virological failure [21–23]. All together, these findings suggest that the virological potency of these generic formulations is at least as high as that of the patent drugs and that treatment with generic antiretrovirals does not increase the risk of virological failure.

We also found similar rates of treatment interruption in the two groups of patients; furthermore, discontinuations for toxicity did not differ between groups and discontinuation of only the generic drug was very uncommon. Taken together, these finding do not support safety concerns about generic drugs.

Indeed, rates of treatment discontinuation were in general quite high, but consistent with those observed in HIV-infected patients in Italy: in the ICONA cohort, 1389/4052 (34.3%) patients stopped their ART over a median follow-up of 12 months, mainly for treatment simplification (29%) [24].

The outcomes of generic antiretrovirals have been investigated in different studied with different design. The only available comparative study was performed in 14 736 patients in Zambia, 49% who initiated a generic formulation of ZDV and 51% initiated a proprietary formulation. No difference in post-90-day mortality was observed between the two groups and no longitudinal differences were detected between formulations also for CD4+ response, weight change and hemoglobin concentration [12]. However, the study was not randomized, groups were not matched and virological response not studied.

All of the other studies on generic antiretrovirals were non-comparative. Generic zidovudine (in fixed dose combination with lamivudine and nevirapine) was studied also in roughly 250 antiretroviral-naïve HIV-1-infected Indian patients [13] and in 109 HIV-1-infected patients living in Cameroon [19]. In the first study, there was an improvement in CD4+ counts over 2 years, but information on virological outcome was not reported; in the second one, 86.9% of the intention-to-treat population had viral load <400 copies/ml at 12 months, CD4+ count had increased by a median of 106 cells/μL at 12 months and drug resistance rarely emerged (incidence rate 3.2 per 100 person-years).

Laurent and coll. reported results of 60 patients followed in an open-label, 24-week multicenter trial in Cameroon during treatment with a generic fixed-dose combination of nevirapine, stavudine, and lamivudine. The proportion of patients with <400 HIV-RNA copies/mL after 24 weeks of treatment was 80% (95% CI: 68%-89%). The median (IQR) change in CD4+ count was 83 (40–178) cells/μL. The probability of remaining alive or free of new AIDS-defining events was 0.85 (95% CI: 0.73%-0.92%). Frequency of disease progression was 32.0 (95% CI: 16.6%-61.5%), while severe adverse effects occurred in 17.8 (7.4–42.7) per 100 person-years, and genotypic resistance mutations in 7.1 (1.8–28.4) per 100 person-years [14].

Similarly, in an open-label combined prospective and retrospective study involving 102 HIV infected patients, treated with a generic fixed-dose combination of nevirapine, stavudine, and lamivudine for 48 weeks, the median CD4+ cell count increased was of 191 cells/μL and 63.7% (intention-to treat analysis) attained <50 HIV-RNA copies/ml [15]. The same regimen was tested also in a 12 months study of 152 adult treatment naive HIV-infected patients. Adverse effects included hypercholesterolemia (43.2%), lipodystrophy (35.5%), hypertriglyceridemia (25%), hypertension (13.1%), peripheral neuropathy (11.9%), hyperlactatemia (2.6%) and lactic acidosis (1.3%) [16]. Virological response not reported in this study.

In a large observational cohort from 21 Médecins Sans Frontieres (MSF) HIV/AIDS programmes taking a generic fixed-dose combination of nevirapine, stavudine, and lamivudine, 6861 patients, after 1 year of follow-up 77% remained on HAART, 91% of these still on the fixed-dose combination regimen [17]. However, also in this case no virological outcome reported.

In a retrospective cohort study of 204 ART-naïve HIV-infected patients were initiated a generic fixed-dose combination of nevirapine, stavudine, and lamivudine; patients were categorized into 2 groups according to the baseline CD4+ count (group A: <50 cell/μL and group B: > or = 50 cell/μL). In the intention-to-treat analysis at 48 weeks, 71.7% (86/120) of group A and 75.0% (63/84) of group B achieved plasma HIV-RNA <50 copies/mL (P = 0.633). At 48 weeks of ART, mean CD4+ were 201 cells/μL in group A and 367 cells/μL in group B, respectively. There were no differences of probabilities to achieve <50 HIV-RNA copies/mL (P = 0.947) and CD4+ increment at 48 weeks between the two groups (P = 0.870) [25].

Finally, in a prospective, multicenter study in China, 198 antiretroviral-naïve HIV-1 positive subjects were randomized to start three nevirapine-based antiretroviral treatments: group A, nevirapine plus zidovudine and didanosine; group B, nevirapine plus stavudine and lamivudine; group C, nevirapine plus zidovudine and lamivudine. At week 52, suppression of plasma HIV-RNA to less than 50 copies/mL was achieved in more patients in group B and C than in group A (68.2%, 69% vs. 39.7%; p<0.001). However, no comparison with branded formulations of these drugs was performed [26].

The results of our study, although very difficult to compare with those mentioned above because of several differences in study design, endpoints and study population, seem consistent with the current body of knowledge on generic antiretrovirals; however, they are unique as they provide comparative data with a matched population treated with branded drugs and a thorough characterization of the virological response in the two groups; furthermore, endpoints were evaluated for each generic drug.

The main limitations of the present study was the lack of randomization; however, this was not possible because, for institutional decision, all the patients that were receiving drugs available as generic formulation had to be switched to the generic formulation. We tried to overcome this limitation by matching switchers with non-switchers; this allowed us to obtain two cohorts with almost identical clinical characteristics, apart from type of ART. Furthermore, we performed a sensitivity analysis restricted to only patients treated with standard “triple” regimens and the results confirmed that switching to generic compounds did not increase the risk of VF or of treatment discontinuation. It must be also underlined that, although we were unable to compare identical regimens, all patients were virologically suppressed at baseline, were on a stable ART regimen and, indeed, those switched to generic formulations continued the same treatment they were receiving at baseline: in fact (as it happened for those who did not switch to generic formulations), the single antiretroviral compounds they were receiving remained exactly the same, which attenuates the differences between the two groups in terms of ART.

Despite these limitations, the results of our study provide unique comparative data and unique information on the efficacy and safety of generic antiretrovirals.

In conclusion, we found no evidence of increased risk of reduced efficacy or increased toxicity after switching from branded to generic lamivudine or zidovudine or efavirenz, after more than one year of follow-up.

## Supporting information

S1 Raw Data (PDF)
