# Supplementary material for: Efficacy and safety of switching from branded to generic antiretrovirals in virologically suppressed HIV-infected patients
Source: PLoS One. 2017 Aug 1;12(8):e0182007. doi: 10.1371/journal.pone.0182007 (PMC5538668; doi:10.1371/journal.pone.0182007)
Supplement: S1 Raw Data — (PDF) [file pone.0182007.s001.pdf]

OK	ID	AVATAR	passaggio	SWITCH	FARMACO	D_HIV	D_ARV	D_AIDS	HCV	HBV	NADIR_CD4	D_SCR	VL_BL	CD4_BL	D_VL_FIRST	VL_FIRST	D_VL_SECOND	VL_SECOND	D_VL_LAST	VL_LAST	CD4_LAST	TIME_UVL
0	0	01/01/2000	9	01/01/2000	ppp	01/01/2000	01/01/2000	01/01/2000 00:00	9	9	999	01/01/2000	999	999	01/01/2000	999	01/01/2000	999	01/01/2000	999	999	999
1	1	15/10/2014	0	25/08/2014		01/01/1990	12/07/1995		1	0	171	25/08/2014	0,9	1015	10/01/2015	0,9	23/05/2015	0,9	14/08/2015	39	808	312,5
1	4	26/09/2014	1	26/11/2014	3TC_AZT	01/01/1984	14/04/1993	01/12/1994 00:00	1	0	44	26/09/2014	0,9	64	05/02/2015	0,9	18/02/2015	0,9	18/02/2015	0,9	113	84
1	6	30/10/2014	0	16/06/2014		01/01/1989	02/04/1997		0	9	219	16/06/2014	39	624	05/12/2014	0,9	25/05/2015	0,9	02/12/2015	55	929	352,5
1	18	23/10/2014	0	04/09/2014		01/01/1988	01/01/1990	19/04/1996 00:00	0	0	130	04/09/2014	0,9	708	12/03/2015	0,9	15/07/2015	0,9	15/07/2015	0,9	549	314
2	22	22/09/2014	1	03/11/2014	3TC	01/01/1985	14/01/1993		1	9	128	22/09/2014	0,9	811	29/01/2015	0,9	28/05/2015	0,9	11/02/2016	0,9	777	465
1	37	15/10/2014	1	15/10/2014	3TC	01/03/1992	15/05/1992	17/07/1992 00:00	0	1	188	15/10/2014	0,9	829	30/12/2014	0,9	27/04/2015	0,9	12/10/2015	0,9	557	362
1	43	23/10/2014	0	30/09/2014		01/11/1991	01/01/1996		0	0	60	30/09/2014	0,9	590	03/04/2015	0,9	06/10/2015	0,9	06/10/2015	0,9	618	371
1	66	03/12/2014	0	16/05/2014		01/01/1986	15/05/1993	01/02/1993 00:00	1	0	53	16/05/2014	0,9	475	12/11/2014	0,9	27/03/2015	0,9	15/01/2016	0,9	624	532
1	75	11/07/2014	1	08/10/2014	3TC	01/11/1990	15/03/1996		0	9	374	11/07/2014	0,9	1481	15/11/2014	0,9	08/04/2015	0,9	20/01/2016	0,9	2011	469
1	82	20/10/2014	0	28/07/2014		01/01/1992	24/04/1992		0	0	167	28/07/2014	0,9	908	09/02/2015	39	17/07/2015	0,9	02/02/2016	0,9	1036	377
1	83	11/08/2014	1	08/10/2014	3TC	01/01/1986	24/02/1993	27/07/2011 00:00	1	0	122	11/08/2014	0,9	318	16/12/2014	0,9	16/04/2015	0,9	09/02/2016	0,9	398	489
1	107	23/10/2014	0	30/09/2014		01/01/1987	01/12/1992		1	0	342	30/09/2014	0,9	889	18/03/2015	50	11/06/2015	48	07/01/2016	39	1067	84,5
1	109	07/08/2014	1	16/10/2014	3TC	01/05/1985	01/11/1989		1	0	25	07/08/2014	0,9	159	13/11/2014	0,9	19/02/2015	0,9	19/02/2015	0,9	132	126
1	118	06/10/2014	1	23/10/2014	3TC	01/01/1992	22/04/1992		0	9	132	06/10/2014	0,9	666	30/03/2015	0,9			30/03/2015	0,9	870	158
1	121	19/11/2014	0	25/06/2014		01/04/1987	01/10/1995		0	0	36	25/06/2014	0,9	529	24/11/2014	39	15/12/2014	39	08/02/2016	39	559	356
1	139	02/10/2014	0	03/09/2014		01/10/1992	04/11/1997		0	0	45	03/09/2014	39	540	09/02/2015	0,9	04/06/2015	39	11/12/2015	0,9	645	258,5
2	151	27/05/2014	1	22/10/2014	3TC	01/10/1992	04/12/1992		9	9	214	27/05/2014	0,9	608	18/11/2014	0,9	27/05/2015	0,9	04/12/2015	0,9	947	408
1	160	25/11/2014	0	16/09/2014		23/07/1992	02/04/1993		0	0	258	16/09/2014	0,9	830								0
2	166	28/08/2014	1	17/11/2014	3TC	01/01/1986	01/01/1991		1	9	228	28/08/2014	0,9	890	08/01/2015	0,9	24/04/2015	39	05/10/2015	0,9	1013	187
1	188	24/07/2014	1	17/11/2014	EFV	01/07/1992	02/03/2009		0	0	340	24/07/2014	0,9	710	02/03/2015	0,9	18/09/2015	0,9	18/09/2015	0,9	524	305
1	189	04/07/2014	1	18/11/2014	3TC_AZT	01/01/1984	07/06/1993	11/12/1992 00:00	1	9	282	04/07/2014	0,9	736	10/03/2015	0,9	18/05/2015	0,9	04/12/2015	0,9	829	381
1	211	07/10/2014	1	07/10/2014	3TC	01/06/1990	29/11/1991	29/11/1991 00:00	9	9	270	07/10/2014	0,9	984	08/04/2015	0,9	03/07/2015	0,9	09/12/2015	0,9	1347	428
1	216	15/09/2014	1	10/11/2014	3TC	01/01/1986	01/02/1989	01/02/1993 00:00	1	1	6	15/09/2014	0,9	599	28/01/2015	0,9	13/05/2015	0,9	14/01/2016	0,9	412	439,5
1	232	02/10/2014	1	23/10/2014	3TC	01/02/1990	19/09/1994		0	0	428	02/10/2014	0,9	775	09/02/2015	0,9	08/06/2015	0,9	01/02/2016	0,9	977	466
1	241	27/10/2014	1	27/10/2014	3TC	01/01/1994	16/02/1996		1	0	192	27/10/2014	0,9	736	08/06/2015	0,9	28/11/2015	0,9	28/11/2015	0,9	828	397
1	244	29/08/2014	1	10/12/2014	3TC	01/03/1993	05/11/1997	01/01/1998 00:00	0	0	325	29/08/2014	39	1582	07/01/2015	0,9	04/05/2015	0,9	28/09/2015	0,9	1449	278
1	249	05/09/2014	1	23/10/2014	EFV	31/03/1993	06/09/2004		0	9	334	05/09/2014	0,9	750	31/10/2014	0,9			31/10/2014	0,9	814	8
1	271	09/10/2014	1	09/10/2014	3TC	15/02/1993	26/06/2005		0	0	185	09/10/2014	0,9	1509								0
1	273	02/10/2014	0	22/09/2014		01/01/1986	08/03/1993		0	0	131	22/09/2014	0,9	660	07/02/2015	0,9	29/08/2015	0,9	23/01/2016	0,9	748	488
2	298	09/10/2014	1	20/10/2014	3TC_AZT	01/08/1992	17/03/1993		9	9	343	09/10/2014	0,9	476	07/04/2015	0,9	15/10/2015	0,9	15/10/2015	0,9	530	360
1	306	07/10/2014	0	21/07/2014		01/01/1992	07/09/1998		9	9	224	21/07/2014	0,9	892	30/12/2014	0,9			30/12/2014	0,9	576	162
1	309	27/08/2014	1	05/11/2014	3TC	01/12/1992	16/03/1998		0	0	317	27/08/2014	39	714	13/02/2015	39	28/08/2015	39	28/08/2015	39	621	0
1	325	29/09/2014	1	02/01/2015	EFV	01/01/1985	27/11/1996	01/03/2000 00:00	1	9	29	29/09/2014	0,9	425	19/01/2015	0,9	01/06/2015	0,9	01/06/2015	0,9	792	150
1	331	17/10/2014	0	17/04/2014		01/01/1989	24/02/1992		1	1	451	17/04/2014	0,9	1166	16/10/2014	0,9	26/03/2015	0,9	24/09/2015	0,9	1350	525
1	368	29/10/2014	0	21/07/2014		01/01/1986	11/01/1993	01/10/1996 00:00	9	9	75	21/07/2014	0,9	714	15/11/2014	39	17/03/2015	0,9	10/08/2015	0,9	560	265,5
1	436	06/11/2014	0	29/09/2014		12/03/1992	20/05/1992	12/03/1992 00:00	1	1	9	29/09/2014	0,9	909	07/05/2015	476775	11/11/2015	39	11/11/2015	39	623	110
1	439	19/09/2014	1	30/10/2014	3TC_AZT	01/01/1987	15/03/1993	20/12/1993 00:00	0	0	53	19/09/2014	0,9	858	14/01/2015	39	29/05/2015	0,9	04/12/2015	0,9	1011	294,5
1	446	22/08/2014	1	06/11/2014	3TC	01/01/1984	01/01/2001		1	1	108	22/08/2014	39	471	07/02/2015	39			07/02/2015	39	676	0
1	456	05/05/2014	1	29/12/2014	3TC_AZT	01/03/1992	27/05/1992		9	1	320	05/05/2014	0,9	996	14/04/2015	0,9	13/07/2015	0,9	13/07/2015	0,9	1221	196
1	491	25/08/2014	1	31/10/2014	3TC	27/05/1991	01/11/1992	21/09/1992 00:00	0	0	132	25/08/2014	0,9	594	19/12/2014	0,9	14/01/2015	0,9	09/11/2015	0,9	175	374
1	493	05/02/2015	0	13/05/2014		01/01/1987	01/01/1990	11/06/1999 00:00	1	0	36	13/05/2014	39	749	18/11/2014	39			18/11/2014	39	679	0
1	556	02/09/2014	1	11/11/2014	3TC	01/07/1993	23/06/1994		0	0	218	02/09/2014	0,9	1061	26/03/2015	0,9	29/09/2015	0,9	29/09/2015	0,9	1481	322
1	573	27/05/2014	1	16/10/2014	3TC	01/01/1985	24/09/1993		0	9	175	27/05/2014	0,9	1136	24/11/2014	52	05/02/2015	0,9	02/02/2016	49	1229	157
1	594	21/10/2014	1	21/10/2014	3TC	01/01/1992	22/01/1997		0	0	283	21/10/2014	0,9	779								0
1	600	02/08/2014	1	17/11/2014	3TC	01/01/1985	15/10/1993		1	0	172	02/08/2014	0,9	537	13/12/2014	0,9	27/03/2015	0,9	27/03/2015	0,9	585	130
3	611	08/10/2014	0	18/09/2014		12/07/1993	19/12/1994		1	0	174	18/09/2014	0,9	591	12/01/2015	39	01/06/2015	0,9	12/10/2015	0,9	780	261
1	618	18/06/2014	1	26/09/2014	3TC	01/01/1985	18/02/1992		1	0	207	18/06/2014	39	796	04/11/2014	39	27/06/2015	40	03/10/2015	39	659	0
1	628	17/11/2014	0	11/04/2014		01/12/1989	20/12/2000		0	0	217	11/04/2014	0,9	521	07/10/2014	0,9	22/04/2015	39	21/09/2015	0,9	538	353,5
1	630	30/12/2014	0	24/06/2014		01/01/1990	15/12/1996		0	0	179	24/06/2014	0,9	673	03/10/2014	0,9	14/03/2015	0,9	30/09/2015	0,9	761	463
1	645	27/10/2014	1	07/11/2014	3TC_AZT	01/01/1991	15/03/1991		1	1	165	27/10/2014	0,9	762	25/03/2015	0,9	05/10/2015	0,9	05/10/2015	0,9	924	332
1	649	06/10/2014	0	26/07/2014		01/01/1986	16/07/1992	01/01/1999 00:00	0	1	153	26/07/2014	39	716	04/03/2015	0,9	29/08/2015	39	29/08/2015	39	716	199,5
1	672	18/11/2014	1	24/11/2014	3TC	01/01/1985	01/01/1995		1	0	188	18/11/2014	39	1563	27/04/2015	39	22/05/2015	49	25/01/2016	0,9	832	84
1	676	10/10/2014	0	11/08/2014		15/05/1993	01/03/1997	29/05/1993 00:00	0	0	3	11/08/2014	0,9	814	08/01/2015	39	02/07/2015	39	22/12/2015	39	743	75
9	730	20/11/2014	0	30/04/2014		01/01/1991	01/06/1992	30/04/2014 00:00	1	9	41	30/04/2014	0,9	631	24/11/2014	885	09/05/2015	61	30/01/2016	0,9	709	146
1	731	16/06/2014	1	21/10/2014	3TC	01/04/1993	11/10/1997	16/12/1996 00:00	1	0	211	16/06/2014	0,9	327	11/11/2014	0,9	18/05/2015	0,9	18/06/2015	0,9	351	240
1	794	17/10/2014	0	31/05/2014		01/01/1986	01/03/1997		0	0	389	31/05/2014	0,9	1356	10/01/2015	0,9			10/01/2015	0,9	1125	224
1	821	01/08/2014	1	24/10/2014	EFV	01/12/1993	22/09/1997	22/02/1995 00:00	0	0	147	01/08/2014	39	931	01/12/2014	0,9	30/06/2015	0,9	03/12/2015	39	760	308
2	824	09/05/2014	1	07/10/2014	3TC	01/05/1986	01/07/1992		1	9	189	09/05/2014	0,9	637	23/10/2014	0,9	09/04/2015	0,9	07/07/2015	0,9	781	273
1	829	21/10/2014	0	26/09/2014		16/09/1985	31/10/1997		0	0	339	26/09/2014	0,9	945	04/03/2015	0,9	16/10/2015	0,9	09/02/2016	0,9	926	501
1	862	03/11/2014	0	03/09/2014		01/01/1992	01/04/1997	19/04/1997 00:00	0	0	14	03/09/2014	0,9	562	04/03/2015	0,9	01/09/2015	0,9	01/09/2015	0,9	506	363
1	877	18/08/2014	1	19/11/2014	3TC_AZT	01/08/1992	10/09/1992	01/09/1993 00:00	0	9	17	18/08/2014	0,9	562	08/01/2015	0,9	01/06/2015	0,9	03/11/2015	0,9	554	349
1	890	13/11/2014	1	02/12/2014	3TC_AZT	15/02/1992	16/05/1995		9	9	275	13/11/2014	0,9	1782	04/05/2015	0,9	28/10/2015	0,9	28/10/2015	0,9	1139	330
1	956	06/11/2014	0	25/09/2014		01/01/1985	20/01/1994		1	0	326	25/09/2014	39	663	26/01/2015	0,9			26/01/2015	0,9	862	61,5
2	963	13/08/2014	1	29/10/2014	3TC	01/07/1992	30/06/1993	01/10/1992 00:00	9	9	146	13/08/2014	0,9	526	30/01/2015	0,9	09/09/2015	0,9	19/01/2016	0,9	605	447
1	982	18/08/2014	1	16/10/2014	3TC	03/03/1994	22/01/1996		0	0	341	18/08/2014	0,9	926	03/02/2015	0,9	29/05/2015	0,9	11/11/2015	39	883	308
1	986	10/05/2014	1	15/10/2014	3TC	01/09/1993	02/12/1993		0	0	192	10/05/2014	0,9	452	22/11/2014	0,9			22/11/2014	0,9	387	38
1	990	14/06/2014	1	29/10/2014	3TC_AZT	01/02/1993	01/05/1997		0	0	490	14/06/2014	0,9	898	29/12/2014	0,9	30/05/2015	0,9	31/10/2015	39	1102	290
1	1025	12/06/2014	1	17/11/2014	EFV	01/01/1985	01/01/1998	01/09/1989 00:00	1	0	180	12/06/2014	0,9	740	29/12/2014	0,9	13/06/2015	0,9	28/12/2015	0,9	623	406
1	1027	20/06/2014	1	22/10/2014	3TC	28/06/1994	15/09/1994	01/06/1994 00:00	0	0	8	20/06/2014	39	218	07/11/2014	0,9	23/02/2015	0,9	19/01/2016	0,9	197	446
1	1043	28/11/2014	1	28/11/2014	3TC	01/01/1993	29/09/1994	15/10/1995 00:00	1	0	227	28/11/2014	39	1325	15/04/2015	115	16/09/2015	39	12/02/2016	0,9	1246	74,5
1	1047	22/09/2014	1	10/11/2014	3TC	13/07/1994	15/12/1996		0	0	341	22/09/2014	0,9	589	20/04/2015	0,9	02/10/2015	47	02/10/2015	47		243,5
1	1048	27/08/2014	1	07/11/2014	3TC	01/09/1989	01/07/1995		1	0	117	27/08/2014	0,9	728								0
3	1053	29/10/2014	0	22/02/2014		01/04/1994	06/11/1995		9	9	183	22/02/2014	0,9	355	11/10/2014	0,9	10/03/2015	0,9	20/08/2015	0,9	452	544
1	1061	26/09/2014	0	12/05/2014		01/01/1994	20/12/1994	20/05/1996 00:00	0	9	6	12/05/2014	0,9	733	20/10/2014	0,9	30/04/2015	0,9	05/11/2015	0,9	749	542
1	1062	29/08/2014	1	29/10/2014	3TC_AZT	01/08/1993	19/10/1993		9	9	160	29/08/2014	0,9	643	06/03/2015	0,9	01/09/2015	0,9	01/09/2015	0,9	629	307
1	1082	10/10/2014	0	27/05/2014		01/11/1992	24/02/1997	12/07/2002 00:00	0	1	295	27/05/2014	0,9	635	20/10/2014	39	07/04/2015	39	07/04/2015	39	658	73
9	1084	06/11/2014	0	01/09/2014		01/01/1995	28/04/1995		0	9	264	01/09/2014	0,9	1110	02/03/2015	0,9	22/09/2015	0,9	22/09/2015	0,9	1002	386
1	1088	12/11/2014	0	30/04/2014		01/08/1995	16/04/1996	01/11/2006 00:00	0	9	44	30/04/2014	0,9	493	13/10/2014	0,9	27/07/2015	0,9	08/01/2016	2341	578	535,5
1	1095	03/10/2014	1	05/12/2014	3TC	01/01/1990	04/11/1992		9	1	19	03/10/2014	0,9	408	20/02/2015	0,9	24/06/2015	0,9	10/12/2015	0,9	444	370
1	1100	08/09/2014	1	31/10/2014	3TC	01/01/1984	14/02/1996		1	0	109	08/09/2014	0,9	467	06/12/2014	0,9	23/02/2015	0,9	13/03/2015	0,9	392	133
1	1114	16/07/2014	1	10/10/2014	3TC	01/11/1992	09/12/1992		9	9	33	16/07/2014	0,9	272	30/10/2014	0,9	24/03/2015	208	24/07/2015	0,9	309	200
1	1132	31/10/2014	0	01/10/2014		01/01/1987	10/06/1995		1	0	214	01/10/2014	0,9	988	23/03/2015	0,9	30/09/2015	0,9	30/09/2015	0,9	881	364
1	1137	09/10/2014	0	22/09/2014		01/01/1995	04/05/1995	29/05/1997 00:00	9	0	2	22/09/2014	0,9	516	08/05/2015	39	02/11/2015	39	02/11/2015	39	667	114
1	1162	14/10/2014	1	03/11/2014	3TC	01/08/1989	01/01/1991	01/05/2004 00:00	1	0	186	14/10/2014	39	566	17/03/2015	39	02/07/2015	0,9	16/12/2015	39	340	137
[illegible]	[illegible]	[illegible]	[illegible]	[illegible]	[illegible]	[illegible]	[illegible]	[illegible]	[illegible]	[illegible]	[illegible]	[illegible]	[illegible]	[illegible]	[illegible]	[illegible]	[illegible]	[illegible]	[illegible]	[illegible]	[illegible]	[illegible]

ID	D_VL_POS	VL_POS	VF	TF	D_BLIP	VL_BLIP	BLIP	D_FREEZING	INIZIO	FINE	STOP_GENERICO	MOTIVO	TC	ABC	TPV	ATV	AZT	DDI	DRV	DVG	EFV	ETV	FPV	FTC	LPV	MRV	NVP	RAL	RPV	RTV	SQV	T	TDF	NRTI	NNRTI	PI	II	FI
0	01/01/2000 00:00	999	9	9	01/01/2000 00:00	999	999	01/01/2000	01/01/2000	01/01/2000 00:00	ppp	pppp	9	9	9	9	9	9	9	9	9	9	9	9	9	9	9	9	9	9	9	9	9	9	9	9	9	9
1				1				17/02/2016	25/11/2008	14/08/2015 00:00		SIDE	1	1	0	1	0	0	0	0	0	0	0	0	0	0	0	0	0	0	0	0	0	1	0	1	0	0
4				1				17/02/2016	06/04/2009	17/03/2015 00:00	ALL	DRUG_INT	1	0	0	0	1	0	0	0	0	0	1	0	0	0	0	0	0	1	0	0	0	1	0	1	0	0
6					02/12/2015 00:00	55	1	17/02/2016	28/08/2006				1	1	0	1	0	0	0	0	0	0	0	0	0	0	0	0	0	0	0	0	0	1	0	1	0	0
18								17/02/2016	06/06/2012				0	0	0	0	0	0	0	0	0	1	0	0	0	1	0	1	0	0	0	0	0	0	1	0	1	1
22								17/02/2016	24/05/2012				1	0	0	1	0	0	0	0	0	0	0	0	0	0	0	0	0	0	0	0	0	1	0	1	0	0
37								17/02/2016	17/09/2014				1	0	0	0	0	0	0	0	0	0	0	0	0	0	1	1	0	0	0	0	0	1	1	0	1	0
43								17/02/2016	10/06/2008				1	1	0	1	0	0	0	0	0	0	0	0	0	0	0	0	0	1	0	0	0	1	0	1	0	0
66								17/02/2016	29/06/2007				0	0	0	0	0	0	1	0	0	0	0	0	0	1	0	1	0	1	0	0	0	0	0	1	1	1
75								17/02/2016	04/06/2014				1	0	0	0	0	0	1	0	0	0	0	0	0	0	0	0	0	1	0	0	0	1	0	1	0	0
82								17/02/2016	08/06/2012				0	0	0	1	0	0	0	0	0	0	0	0	0	1	0	1	0	0	0	0	0	0	0	1	1	1
83								17/02/2016	23/09/2013				1	0	0	0	0	0	0	0	0	0	0	0	1	0	0	0	0	0	0	0	0	1	0	1	0	0
107					18/03/2015 00:00	50	1	17/02/2016	29/04/2014				1	1	0	1	0	0	0	0	0	0	0	0	0	0	0	0	0	1	0	0	0	1	0	1	0	0
109				1				17/02/2016	03/12/2013	05/06/2015 00:00	ALL	DRUG_INT	1	0	0	0	0	0	0	0	0	0	1	0	0	0	0	0	0	0	0	0	0	1	0	1	0	0
118				1				17/02/2016	17/05/2007	22/04/2015 00:00	ALL	MEDICO	1	0	0	0	0	1	0	0	0	0	0	0	0	0	1	0	0	0	0	0	0	1	1	0	0	0
121								17/02/2016	31/05/2010				0	0	0	0	0	0	1	0	0	1	0	0	0	0	0	1	0	1	0	0	0	0	1	1	1	0
139								17/02/2016	21/12/2011				0	0	0	0	0	0	1	0	0	0	0	1	0	0	0	0	0	1	0	0	1	1	0	1	0	0
151								17/02/2016	12/08/2011				1	0	0	0	0	0	1	0	0	0	0	0	0	0	0	1	0	1	0	0	0	1	0	1	1	0
160				1				17/02/2016	17/11/2009	15/11/2014 00:00		TOXY_OSTEO	0	0	0	1	0	0	0	0	0	0	0	1	0	0	0	0	0	1	0	0	1	1	0	1	0	0
166								17/02/2016	13/11/2007				1	0	0	0	0	0	0	0	0	0	1	0	0	0	0	1	0	0	0	0	0	1	0	1	1	0
188								17/02/2016	23/05/2011				1	1	0	0	0	0	0	0	1	0	0	0	0	0	0	0	0	0	0	0	0	1	1	0	0	0
189								17/02/2016	04/05/2009				1	0	0	0	1	0	0	0	0	0	0	0	0	0	1	0	0	0	0	0	0	1	1	0	0	0
211								17/02/2016	01/09/2009				1	0	0	1	0	0	0	0	0	0	0	0	0	0	0	1	0	0	0	0	0	1	0	1	1	0
216								17/02/2016	22/01/2013				1	0	0	1	0	0	0	0	0	0	0	0	0	0	0	1	0	0	0	0	0	1	0	1	1	0
232								17/02/2016	14/06/2012				1	0	0	1	0	0	0	0	0	0	0	0	0	0	0	0	0	1	0	0	0	1	0	1	0	0
241								17/02/2016	12/04/2013				1	0	0	1	0	0	0	0	0	0	0	0	0	0	0	0	0	1	0	0	0	1	0	1	0	0
244								17/02/2016	24/02/2011				1	0	0	1	0	0	0	0	0	0	0	0	0	0	0	0	0	1	0	0	0	1	0	1	0	0
249				1				17/02/2016	29/09/2014	24/11/2014 00:00	EFV	TOXY_NERV	1	1	0	0	0	0	0	0	1	0	0	0	0	0	0	0	0	0	0	0	0	1	1	0	0	0
271				1				17/02/2016	01/02/2005	28/10/2014 00:00	ALL	SEMPLIFICAZIONE	1	0	0	1	0	1	0	0	0	0	0	0	0	0	0	0	0	0	0	0	0	1	0	1	0	0
273								17/02/2016	05/06/2013				0	0	0	0	0	0	1	0	0	0	0	1	0	0	0	0	0	1	0	0	1	1	0	1	0	0
298				1				17/02/2016	30/04/2007	19/10/2015 00:00	COMBI	SEMPLIFICAZIONE	1	0	0	1	1	0	0	0	0	0	0	0	0	0	0	0	0	0	0	0	0	1	0	1	0	0
306				1				17/02/2016	06/12/2010	05/01/2015 00:00		DISLIPIDEMIA	0	0	0	0	0	0	1	0	0	0	0	1	0	0	0	0	0	1	0	0	1	1	0	1	0	0
309				1				17/02/2016	24/12/2013	30/09/2015 00:00	ALL	MORTE	1	0	0	1	0	0	0	0	0	0	0	0	0	0	0	0	0	0	0	0	0	1	0	1	0	0
325				1				17/02/2016	27/02/2009	19/06/2015 00:00	NO	SIDE	0	0	1	0	0	0	0	0	1	0	0	1	0	0	0	0	0	1	0	0	1	1	1	1	0	0
331								17/02/2016	28/12/2009				1	1	0	1	0	0	0	0	0	0	0	0	0	0	0	0	0	0	0	0	0	1	0	1	0	0
368				1				17/02/2016	02/03/2009	31/08/2015 00:00		SEMPLIFICAZIONE	0	0	0	0	0	0	1	0	0	0	0	0	0	0	0	1	0	1	0	0	1	1	0	1	1	0
436					07/05/2015 00:00	476775	1	17/02/2016	30/03/2011				0	0	0	0	0	0	1	0	0	0	0	1	0	0	0	0	0	1	0	0	1	1	0	1	0	0
439								17/02/2016	13/06/2011				1	0	0	0	1	0	1	0	0	0	0	0	0	0	0	0	0	1	0	0	0	1	0	1	0	0
446				1				17/02/2016	23/09/2009	16/07/2015 00:00	NO	DRUG_INT	1	0	0	0	0	0	1	0	0	0	0	0	0	1	0	1	0	1	0	0	0	1	0	1	1	1
456				1				17/02/2016	31/03/2014	03/08/2015 00:00	COMBI	ABNORMAL FAT	1	0	0	0	1	0	0	0	0	0	0	0	0	0	0	1	0	0	0	0	0	1	0	0	1	0
491								17/02/2016	21/01/2013				1	0	0	0	0	0	1	0	0	0	0	0	0	0	0	0	0	1	0	0	0	1	0	1	0	0
493				1				17/02/2016	11/05/2011	02/12/2014 00:00		TOXY_OSTEO	0	0	0	0	0	0	1	0	0	0	0	1	0	0	0	0	0	1	0	0	1	1	0	1	0	0
556								17/02/2016	20/02/2013				1	0	0	1	0	0	0	0	0	0	0	0	0	0	1	0	0	0	0	0	0	1	1	1	0	0
573					24/11/2014 00:00	52	1	17/02/2016	24/08/2012				1	0	0	0	0	0	1	0	0	0	0	0	0	0	0	1	0	1	0	0	0	1	0	1	1	0
594				1				17/02/2016	03/07/2013	11/11/2014 00:00	NO	TOXY_RENALE	1	0	0	1	0	0	0	0	0	0	0	0	0	0	0	0	0	1	0	0	0	1	0	1	0	0
600				1				17/02/2016	19/12/2011	27/03/2015 00:00	ALL	COMORBIDITY	1	0	0	1	0	0	0	0	0	0	0	0	0	1	0	0	0	0	0	0	0	1	0	1	0	1
611								17/02/2016	30/08/2012				0	0	0	0	0	0	1	0	0	0	0	1	0	0	0	0	0	1	0	0	1	1	0	1	0	0
618								17/02/2016	27/06/2014				1	0	0	0	0	0	1	0	0	0	0	0	0	0	0	0	0	1	0	0	0	1	0	1	0	0
628								17/02/2016	27/03/2012				0	0	0	1	0	0	0	0	0	0	0	0	0	1	0	0	0	1	0	0	0	0	0	1	0	1
630								17/02/2016	03/04/2014				0	0	0	0	0	0	1	0	0	0	0	1	0	1	0	0	0	1	0	0	1	1	0	1	0	1
645								17/02/2016	03/11/2006				1	0	0	0	1	0	0	0	0	0	0	0	0	0	0	0	0	0	0	0	1	1	0	0	0	0
649								17/02/2016	28/07/2008				0	0	0	0	0	0	0	0	1	0	0	1	0	0	0	0	0	0	0	0	1	1	1	0	0	0
672					07/09/2015 00:00	143	1	17/02/2016	21/02/2012				1	0	0	0	0	0	1	0	0	1	0	0	0	0	0	0	0	1	0	0	0	1	1	1	0	0
676								17/02/2016	19/03/2007				1	1	0	0	0	0	0	0	0	0	0	0	1	0	0	0	0	0	0	0	0	1	0	1	0	0
730	24/11/2014 00:00	885	1					17/02/2016	24/04/2006				0	0	0	0	0	0	0	1	0	0	0	1	1	0	0	0	0	0	0	0	1	1	0	1	1	0
731				1				17/02/2016	20/12/2013	17/10/2015 00:00	NO	COMORBIDITY	1	0	0	1	0	0	0	0	0	0	0	0	0	0	0	0	0	0	0	0	0	1	0	1	0	0
794				1				17/02/2016	05/11/2007	26/01/2015 00:00		SEMPLIFICAZIONE	0	0	0	0	0	0	0	0	0	0	0	1	1	0	0	0	0	0	0	0	1	1	0	1	0	0
821								17/02/2016	18/06/2012				0	0	0	0	0	0	0	0	1	0	0	0	0	1	0	1	0	0	0	0	0	0	1	0	1	1
824				1				17/02/2016	18/11/2013	27/07/2015 00:00	NO	SEMPLIFICAZIONE	1	0	0	1	0	0	0	0	0	0	0	0	0	0	0	0	0	0	0	0	0	1	0	1	0	0
829								17/02/2016	13/03/2013				0	0	0	1	0	0	0	0	0	0	0	1	0	0	0	0	0	1	0	0	0	1	0	1	0	0
862								17/02/2016	08/07/2008				1	1	0	0	0	0	0	0	0	0	0	0	0	0	1	0	0	0	0	0	0	1	1	0	0	0
877								17/02/2016	24/09/2006				1	0	0	1	1	0	0	0	0	0	0	0	0	0	0	0	0	0	0	0	0	1	0	1	0	0
890								17/02/2016	05/10/2000				1	0	0	0	1	0	0	0	0	0	0	0	0	0	1	0	0	0	0	0	0	1	1	0	0	0
956				1				17/02/2016	27/09/2013	16/03/2015 00:00		DRUG_INT	0	0	0	0	0	0	0	0	0	1	0	1	0	0	0	1	0	0	0	0	1	1	1	0	1	0
963								17/02/2016	27/03/2010				1	0	0	0	0	0	0	0	0	0	0	0	0	0	0	0	0	0	0	0	1	1	0	0	0	0
982								17/02/2016	08/03/2012				1	0	0	1	0	0	0	0	0	0	0	0	0	0	0	0	0	1	0	0	0	1	0	1	0	0
986				1				17/02/2016	06/11/2009	20/12/2014 00:00	NO	SEMPLIFICAZIONE	1	0	0	0	0	0	1	0	0	0	0	0	0	0	0	1	0	1	0	0	0	1	0	1	1	0
990								17/02/2016	30/04/2008				1	0	0	0	1	0	0	0	0	0	0	0	0	0	1	0	0	0	0	0	0	1	1	0	0	0
1025								17/02/2016	19/10/2011				0	0	0	1	0	0	0	0	1	0	0	1	0	0	0	0	0	0	0	0	0	1	1	1	0	0
1027								17/02/2016	18/05/2013				1	0	0	0	0	0	1	0	0	0	0	0	0	0	0	1	0	1	0	0	0	1	0	1	1	0
1043					15/04/2015 00:00	115	1	17/02/2016	14/05/2005				1	0	0	1	0	0	0	0	0	0	0	0	0	0	0	0	0	1	0	0	1	1	0	1	0	0
1047								17/02/2016	03/06/2013				1	0	0	1	0	0	0	0	0	0	0	0	0	0	0	0	0	0	0	0	0	1	0	1	0	0
1048				1				17/02/2016	06/05/2008	07/02/2015 00:00	ALL	MORTE	1	0	0	0	0	0	0	0	0	0	0	0	1	0	0	0	0	0	0	0	0	1	0	1	0	0
1053				1				17/02/2016	27/01/2010	03/11/2015 00:00		TOXY_RENALE	0	0	0	0	0	0	0	0	0	0	1	1	0	0	0	0	0	1	0	0	1	1	0	1	0	0
1061				1				17/02/2016	21/02/2011	23/11/2015 00:00		CONCERN_CARDIO	1	1	0	0	0	0	1	0	0	0	0	0	0	0	0	0	0	1	0	0	0	1	0	1	0	0
1062								17/02/2016	05/03/2010				1	0	0	0	1	0	0	0	0	0	0	0	0	0	1	0	0	0	0	0	0	1	1	0	0	0
1082				1				17/02/2016	27/01/2011	20/04/2015 00:00		SEMPLIFICAZIONE	0	0	0	1	0	1	0	0	0	0	0	0	0	0	1	0	0	1	0	0	0	1	1	1	0	0
1084								17/02/2016	17/11/2005				0	0	0	0	0	0	0	0	0	0	0	1	0	0	1	0	0	0	0	0	1	1	1	0	0	0
1088					08/01/2016 00:00	2341	1	17/02/2016	22/09/2008				0	0	0	0	0	0	1	0	0	0	0	1	0	0	0	0	0	1	0	0	1	1	0	1	0	0
1095								17/02/2016	29/06/2012				1	0	0	0	0	0	1	0	0	0	0	0	0	0	0	0	0	1	0	0	0	1	0	1	0	0
1100				1				17/02/2016	05/02/2013	21/04/2015 00:00	NO	DRUG_INT	1	0	0	1	0	0	0	0	0	0	0	0	0	0	0	0	0	1	0	0	0	1	0	1	0	0
1114				1	24/03/2015 00:00	208	1	17/02/2016	29/07/2014	07/08/2015 00:00	NO	TOXY_RENALE	1	0	0	1	0	0	0	0	0	0	0	0	0	0	0	0	0	1	0	0	0	1	0	1	0	0
1132								17/02/2016	26/05/2011				0	0	0	1	0	0	0	0	0	0	0	0	0	0	1	0	1	0	0	0	0	0	0	1	1	0
1137								17/02/2016	06/02/2012				0	0	0	0	0	0	1	0	0	0	0	0	0	0	1	0	1	0	0	0	0	0	0	1	1	0
1162								17/02/2016	03/02/2012				1	0	0	0	0	0	0	0	0	1	0	0	0	0	0	1	0	0	0	0	0	1	1	0	1	0
[illegible]	[illegible]	[illegible]	[illegible]	[illegible]	[illegible]	[illegible]	[illegible]	[illegible]	[illegible]	[illegible]	[illegible]	[illegible]	[illegible]	[illegible]	[illegible]	[illegible]	[illegible]	[illegible]	[illegible]	[illegible]	[illegible]	[illegible]	[illegible]	[illegible]	[illegible]	[illegible]	[illegible]	[illegible]	[illegible]	[illegible]	[illegible]	[illegible]	[illegible]	[illegible]	[illegible]	[illegible]	[illegible]	[illegible]

1	2437	13/11/2014	1	28/11/2014	3TC	01/07/1991	15/05/2000		1	0	451	13/11/2014	0,9	1395	[illegible]
1	2443	27/10/2014	1	03/02/2015	3TC	01/05/1998	15/07/1998	29/05/1998 00:00	1	0	10	27/10/2014	0,9	224	[illegible]
1	2458	24/12/2014	1	24/12/2014	EFV	01/05/1998	24/06/1998		1	0	142	24/12/2014	0,9	1043	[illegible]
1	2470	21/10/2014	1	17/12/2014	EFV	18/06/1999	18/06/1999	17/06/1999 00:00	0	0	32	21/10/2014	0,9	515	[illegible]
1	2472	21/10/2014	0	19/09/2014		01/01/1992	27/04/1998		1	0	138	19/09/2014	43	626	[illegible]
1	2485	02/12/2014	0	21/07/2014		01/01/1997	23/05/1998	01/01/1997 00:00	0	0	327	21/07/2014	39	327	[illegible]
1	2486	12/09/2014	1	09/12/2014	3TC	01/01/1989	15/12/1997		0	0	244	12/09/2014	0,9	854	[illegible]
1	2495	17/10/2014	0	30/05/2014		01/01/1991	02/02/1999		0	0	298	30/05/2014	0,9	663	[illegible]
1	2497	31/10/2014	0	13/05/2014		01/01/1991	01/01/1991	27/10/1998 00:00	0	0	158	13/05/2014	0,9	880	[illegible]
1	2503	21/10/2014	0	29/08/2014		01/01/1988	21/12/1996	01/10/1996 00:00	1	0	5	29/08/2014	0,9	184	[illegible]
1	2504	12/11/2014	0	14/03/2014		01/01/1985	01/05/1999		1	9	118	14/03/2014	39	381	[illegible]
9	2505	03/11/2014	0	01/09/2014		01/01/1988	01/06/1995		1	0	157	01/09/2014	0,9	833	[illegible]
1	2512	10/10/2014	0	12/09/2014		01/06/1996	15/06/1996		9	9	32	12/09/2014	0,9	155	[illegible]
1	2516	10/11/2014	0	02/05/2014		01/01/1987	15/05/1994	10/07/1997 00:00	1	0	80	02/05/2014	0,9	759	[illegible]
9	2522	10/10/2014	0	19/09/2014		01/01/1987	10/12/2009		1	0	268	19/09/2014	0,9	1021	[illegible]
2	2523	25/09/2014	1	20/11/2014	3TC	01/03/1996	15/08/1996		0	0	264	25/09/2014	0,9	1082	[illegible]
1	2532	24/10/2014	0	04/06/2014		01/01/1986	01/01/1999	11/11/1998 00:00	0	0	155	04/06/2014	39	863	[illegible]
1	2548	23/09/2014	1	06/10/2014	3TC	01/12/1989	01/06/1990		0	0	186	23/09/2014	0,9	688	[illegible]
1	2551	24/12/2014	0	17/05/2014		13/07/1999	22/09/1999	23/06/1999 00:00	1	1	111	17/05/2014	39	1069	[illegible]
1	2553	29/05/2014	1	21/10/2014	3TC_AZT	01/01/1990	10/02/1998		1	0	413	29/05/2014	0,9	870	[illegible]
1	2557	11/07/2014	1	21/11/2014	3TC	01/01/1988	01/10/1998		1	0	49	11/07/2014	0,9	425	[illegible]
9	2562	20/11/2014	0	12/11/2014		01/11/1991	01/03/1998		0	0	298	12/11/2014	39	1033	[illegible]
1	2564	16/10/2014	0	27/09/2014		01/05/1998	24/06/1998		0	1	209	27/09/2014	0,9	1135	[illegible]
9	2566	17/03/2015	0	02/12/2014		05/06/1998	30/06/1998		0	0	181	02/12/2014	0,9	1210	[illegible]
1	2571	31/10/2014	0	02/04/2014		01/01/1991	03/03/1999	03/07/2009 00:00	0	0	278	02/04/2014	39	982	[illegible]
1	2572	17/12/2014	0	06/09/2014		01/01/1991	01/05/1993	01/01/1995 00:00	0	0	30	06/09/2014	0,9	633	[illegible]
1	2576	01/09/2014	1	22/10/2014	EFV	01/02/1998	01/02/1998	11/02/1998 00:00	0	0	10	01/09/2014	39	272	[illegible]
1	2577	28/11/2014	0	12/03/2014		01/02/1997	01/05/1997	01/05/1997 00:00	1	0	393	12/03/2014	0,9	1353	[illegible]
1	2580	04/10/2014	1	31/10/2014	EFV	01/12/1997	15/12/1997	19/12/1997 00:00	1	1	5	04/10/2014	0,9	909	[illegible]
1	2588	31/07/2014	1	10/11/2014	3TC_AZT	01/09/1998	10/05/2007	01/04/2012 00:00	0	0	222	31/07/2014	0,9	736	[illegible]
1	2595	03/11/2014	1	03/11/2014	3TC	01/01/1986	01/01/2001		1	0	196	03/11/2014	0,9	798	[illegible]

[illegible]

1	5082	24/10/2014	0	09/06/2014		01/01/1990	01/01/1993		1	0	215	09/06/2014	0,9	880	04/12/2014	0,9	17/02/2015	0,9	01/12/2015	0,9	765	540								17/02/2016	29/10/2010				1	1	0	1	0	0	0	0	0	0	0	0	0	0	0	0	0	1	0	0	0	1	0	1	0	0
1	5100	21/10/2014	0	27/03/2014		01/01/1987	01/01/1989		1	9	166	27/03/2014	0,9	412	05/01/2015	0,9	11/05/2015	0,9	15/09/2015	0,9	745	537								17/02/2016	08/02/2013				0	0	0	0	0	0	0	0	0	0	0	1	0	0	0	1	0	0	0	0	1	1	0	0	1	0
1	5104	26/11/2014	1	26/11/2014	3TC	06/11/2003	15/11/2003		0	0	428	26/11/2014	39	1065	10/04/2015	0,9	20/10/2015	0,9	20/10/2015	0,9	961	260,5								17/02/2016	06/02/2014				1	0	0	0	0	0	0	0	0	0	0	0	0	0	0	0	1	0	0	0	1	1	1	0	0	0
1	5123	03/11/2014	0	05/09/2014		01/01/1995	28/01/1997		1	0	211	05/09/2014	39	776	14/01/2015	41	05/03/2015	39	24/12/2015	0,9		100					03/12/2015 00:00	73	1	17/02/2016	29/09/2011				0	0	0	0	0	0	0	0	0	0	0	1	0	0	0	1	0	0	0	0	1	1	0	0	1	0
1	5124	10/09/2014	1	27/10/2014	3TC	01/01/1993	01/01/1993		0	0	169	10/09/2014	0,9	575	06/02/2015	0,9			06/02/2015	0,9	556	102				1				17/02/2016	04/02/2013	19/02/2015 00:00	NO	CONCERN_CARDIO	1	0	0	0	0	0	1	0	0	0	0	0	0	0	0	0	0	1	0	0	0	1	0	1	0	0
9	5126	08/10/2014	0	20/06/2014		09/12/2003	28/02/2005		0	0	238	20/06/2014	39	724	19/11/2014	0,9	06/05/2015	0,9	23/10/2015	0,9	846	329								17/02/2016	11/11/2009				0	0	0	0	0	0	0	0	1	0	0	1	0	0	0	0	0	0	0	0	1	1	1	0	0	0
1	5127	04/11/2014	1	17/11/2014	3TC_AZT	29/04/2003	08/08/2003		0	0	113	04/11/2014	0,9	386	26/02/2015	0,9			26/02/2015	0,9	392	101								17/02/2016	01/03/2013				1	0	0	0	1	0	0	0	0	0	0	0	0	0	1	0	0	0	0	0	0	1	1	0	0	0
1	5131	04/01/2014	1	10/11/2014	3TC	26/03/2003	01/04/2003		0	0	179	04/01/2014	0,9	652	14/02/2015	39			14/02/2015	39	646	48				1				17/02/2016	15/07/2008	11/04/2015 00:00	ALL	TOXY_OSTEO	1	0	0	1	0	0	0	0	0	0	0	0	0	0	0	0	0	1	0	0	1	1	0	1	0	0
2	5146	22/10/2014	1	02/02/2015	3TC	01/01/1989	15/06/1997		0	0	147	22/10/2014	0,9	886	15/04/2015	0,9	14/09/2015	0,9	14/09/2015	0,9	813	224				1				17/02/2016	19/01/2011	15/12/2015 00:00	NO	DRUG_INT	1	0	0	1	0	0	0	0	0	0	0	0	0	0	0	1	0	1	0	0	0	1	0	1	1	0
1	5158	20/11/2014	0	26/09/2014		01/01/1993	15/06/1993		0	0	150	26/09/2014	0,9	418	10/01/2015	0,9	08/05/2015	99	18/01/2016	676		165	21/12/2015 00:00	534	1		08/05/2015 00:00	99	1	17/02/2016	28/11/2011				1	1	0	0	0	0	0	0	0	0	0	0	0	1	0	1	0	0	0	0	0	1	0	0	1	1
1	5162	26/11/2014	0	05/08/2014		15/01/2004	11/04/2005		1	9	280	05/08/2014	0,9	740	24/10/2014	0,9	28/01/2015	0,9	28/01/2015	0,9	1091	176				1				17/02/2016	19/12/2011	20/03/2015 00:00		DRUG_INT	1	1	0	0	0	0	0	0	0	0	0	0	1	0	0	0	0	0	0	0	0	1	0	1	0	0
1	5172	03/02/2015	0	08/09/2014		01/02/1987	15/01/1996		0	1	378	08/09/2014	0,9	515								0				1				17/02/2016	01/03/2011	31/10/2014 00:00		SEMPLIFICAZIONE	0	0	0	1	0	0	0	0	0	0	0	1	0	0	0	0	0	0	0	0	1	1	0	1	0	0
1	5183	07/11/2014	0	25/09/2014		01/01/1988	01/01/1997		1	0	314	25/09/2014	0,9	1624	09/01/2015	0,9	08/05/2015	39	19/01/2016	104	1088	293,5					19/01/2016 00:00	104	1	17/02/2016	03/01/2007				0	0	0	0	0	0	0	0	0	0	0	1	1	0	0	0	0	0	0	0	1	1	0	1	0	0
1	5191	05/11/2014	1	05/11/2014	3TC	10/04/2004	25/02/2011		0	0	6	05/11/2014	0,9	259	02/05/2015	0,9			02/05/2015	0,9	410	178								17/02/2016	30/05/2012				1	0	0	1	0	0	0	0	0	0	0	0	0	0	0	0	0	1	0	0	0	1	0	1	0	0
1	5194	29/03/2014	1	30/09/2014	DOPPIO	19/02/2004	01/04/2004		0	0	528	29/03/2014	0,9	621	21/10/2014	0,9			21/10/2014	0,9	689	21				1				17/02/2016	01/04/2014	13/02/2015 00:00	ALL	CONCERN_TOXY	1	0	0	0	0	1	0	0	1	0	0	0	0	0	0	0	0	0	0	0	0	1	1	0	0	0
1	5230	27/10/2014	0	10/07/2014		01/01/1989	01/12/1989		0	0	189	10/07/2014	0,9	554	31/10/2014	0,9	09/04/2015	39	11/01/2016	0,9	462	424								17/02/2016	10/09/2009				0	0	0	0	0	0	0	0	0	1	0	0	0	1	0	1	0	0	0	0	0	0	1	0	1	1
1	5231	19/09/2014	1	18/11/2014	3TC	01/02/1992	01/04/1992	01/04/1996 00:00	0	0	2	19/09/2014	0,9	455	20/01/2015	0,9	19/05/2015	0,9	23/09/2015	0,9	338	456								17/02/2016	02/02/2012				1	0	0	1	0	0	0	0	0	0	0	0	0	0	1	0	0	0	0	0	0	1	0	1	1	0
1	5236	29/09/2014	1	13/10/2014	3TC_AZT	01/12/1990	01/11/1996		1	0	432	29/09/2014	0,9	798	19/03/2015	0,9	24/09/2015	0,9	24/09/2015	0,9	956	346								17/02/2016	05/12/2012				1	0	0	0	1	0	0	0	0	0	0	0	0	1	0	0	0	0	0	0	0	1	1	0	0	0
1	5251	03/10/2014	0	09/07/2014		01/01/1985	06/04/2006		1	9	310	09/07/2014	0,9	750	08/01/2015	0,9	09/03/2015	0,9	02/11/2015	0,9	623	481								17/02/2016	04/03/2014				1	1	0	0	0	0	1	0	0	0	0	0	0	0	0	0	0	1	0	0	0	1	0	1	0	0
1	5266	11/06/2014	1	10/10/2014	3TC	01/01/1992	01/12/1998		1	9	626	11/06/2014	0,9	1691	21/10/2014	0,9	27/01/2015	0,9	27/01/2015	0,9	1693	109				1				17/02/2016	04/11/2011	12/03/2015 00:00	NO	DRUG_INT	1	0	0	0	0	0	0	0	0	0	0	0	1	0	0	0	0	0	0	0	0	1	0	1	0	0
1	5273	29/10/2014	0	07/06/2014		30/04/2004	31/05/2004		0	0	415	07/06/2014	48	1186	03/12/2014	89	22/05/2015	39	02/11/2015	39	1339	0					03/12/2014 00:00	89	1	17/02/2016	22/05/2008				0	0	0	0	0	0	0	0	1	0	0	1	0	0	0	0	0	0	0	0	1	1	1	0	0	0
2	5274	28/08/2014	1	12/11/2014	DOPPIO	04/07/2001	04/07/2001		0	0	358	28/08/2014	0,9	456	05/05/2015	0,9	01/10/2015	0,9	07/01/2016	0,9	799	421								17/02/2016	30/01/2009				1	0	0	0	1	0	0	0	1	0	0	0	0	0	0	0	0	0	0	0	0	1	1	0	0	0
1	5279	07/04/2014	1	03/11/2014	EFV	01/01/1999	21/03/2000		1	0	315	07/04/2014	0,9	772	05/05/2015	0,9	02/11/2015	0,9	02/11/2015	0,9	785	364								17/02/2016	24/06/2008				0	0	0	0	0	0	0	0	1	0	0	1	0	0	0	0	0	0	0	0	1	1	1	0	0	0
1	5303	08/09/2014	1	23/10/2014	3TC	01/01/1986	01/01/1988		1	0	458	08/09/2014	0,9	870	22/01/2015	39	02/07/2015	0,9	11/01/2016	0,9	825	319								17/02/2016	05/07/2012				1	0	0	1	0	0	0	0	0	0	0	0	0	0	0	0	0	1	0	0	0	1	0	1	0	0

[illegible]

1	7436	22/08/2014	1	02/10/2014	3TC	01/09/2007	01/09/2008		0	0	304	22/08/2014	0,9	789	31/01/2015	39			31/01/2015	39	887	60,5				1				17/02/2016	11/01/2012	12/02/2015 00:00	ALL	SEMPLIFICAZIONE	1	0	0	1	0	0	0	0	0	0	0	0	0	1	0	0	0	1	0	0	0	1	0	1	0	1
1	7440	09/10/2014	0	22/09/2014		19/03/2007	18/03/2009		0	0	325	22/09/2014	39	1064	07/02/2015	0,9	07/09/2015	0,9	07/09/2015	0,9	1085	281								17/02/2016	01/06/2014				0	0	0	0	0	0	0	0	0	0	0	1	0	0	0	0	1	0	0	0	1	1	1	0	0	0
1	7464	21/10/2014	1	03/12/2014	EFV	01/11/2002	29/10/2010		0	1	420	21/10/2014	0,9	610	27/03/2015	0,9	28/09/2015	0,9	22/01/2016	0,9	494	415				1				17/02/2016	06/05/2011	22/01/2016 00:00	EFV	SIDE	0	0	0	0	0	0	0	0	1	0	0	1	0	0	0	0	0	0	0	0	1	1	1	0	0	0
1	7526	16/07/2014	1	09/10/2014	3TC	01/01/1985	01/01/1999		1	0	286	16/07/2014	0,9	702	07/11/2014	56	10/03/2015	0,9	22/09/2015	0,9	802	272					07/11/2014 00:00	56	1	17/02/2016	21/02/2011				1	0	0	0	0	0	1	0	0	0	0	0	0	0	0	0	0	1	0	0	0	1	0	1	0	0
1	7538	09/10/2014	1	09/10/2014	3TC	10/12/2008	11/11/2010		0	0	352	09/10/2014	0,9	1035	29/01/2015	0,9	26/05/2015	0,9	26/05/2015	0,9	924	229				1				17/02/2016	23/04/2014	23/06/2015 00:00	NO	TOXY_RENALE	1	0	0	1	0	0	0	0	0	0	0	0	0	0	0	0	0	1	0	0	0	1	0	1	0	0
1	7548	02/10/2014	1	07/11/2014	3TC	01/07/2005	25/03/2008		9	9	512	02/10/2014	0,9	995	28/04/2015	0,9	11/11/2015	39	11/11/2015	39	1110	270,5								17/02/2016	15/04/2014				1	0	0	0	0	0	1	0	0	0	0	0	0	0	0	0	0	1	0	0	0	1	0	1	0	0
1	7591	11/12/2014	0	09/09/2014		13/10/2008	14/11/2011		0	0	249	09/09/2014	0,9	653	05/03/2015	39	01/09/2015	0,9	01/09/2015	0,9	706	178,5								17/02/2016	15/10/2013				0	0	0	0	0	0	0	0	0	0	0	1	0	0	0	0	1	0	0	0	1	1	1	0	0	0
1	7621	25/10/2014	1	11/11/2014	EFV	18/01/1999	07/08/2007		0	0	187	25/10/2014	0,9	629	18/04/2015	0,9	21/10/2015	0,9	21/10/2015	0,9	622	344								17/02/2016	18/11/2008				0	0	0	0	0	0	0	0	1	0	0	1	0	0	0	0	0	0	0	0	1	1	1	0	0	0
1	7626	14/05/2014	1	31/10/2014	3TC	01/01/1993	01/06/1996		0	0	157	14/05/2014	0,9	614	26/11/2014	0,9	22/05/2015	0,9	01/12/2015	0,9	703	396								17/02/2016	27/05/2013				1	0	0	0	0	0	1	0	0	0	0	0	0	0	0	1	0	1	0	0	0	1	0	1	1	0
9	7631	25/11/2014	0	02/10/2014		01/10/2008	19/02/2010		0	0	293	02/10/2014	0,9	584	15/04/2015	0,9	30/09/2015	39	30/09/2015	39	530	279								17/02/2016	19/02/2010				0	0	0	0	0	0	0	0	1	0	0	1	0	0	0	0	0	0	0	0	1	1	1	0	0	0
1	7706	21/11/2014	0	14/07/2014		15/01/2009	27/02/2012		0	0	265	14/07/2014	0,9	1038	13/11/2014	0,9			13/11/2014	0,9	939	122				1				17/02/2016	29/01/2013	12/12/2014 00:00		TOXY_NERV	0	0	0	0	0	0	0	0	1	0	0	1	0	0	0	0	0	0	0	0	1	1	1	0	0	0
1	7721	16/10/2014	0	08/07/2014		20/01/2009	02/02/2009	01/01/2009 00:00	0	0	39	08/07/2014	0,9	376	28/10/2014	0,9	18/03/2015	0,9	24/11/2015	0,9	293	504				1				17/02/2016	29/09/2010	11/12/2015 00:00		SEMPLIFICAZIONE	0	0	0	0	0	0	1	0	0	0	0	1	0	0	0	0	0	1	0	0	1	1	0	1	0	0
1	7736	28/11/2014	0	25/06/2014		01/01/1985	01/01/2000		1	0	24	25/06/2014	0,9	372	20/10/2014	0,9			20/10/2014	0,9	525	117				1				17/02/2016	01/08/2005	18/11/2014 00:00		SEMPLIFICAZIONE	0	0	0	1	0	0	0	0	0	0	0	1	0	0	0	0	0	1	0	0	1	1	0	1	0	0
1	7741	18/11/2014	0	05/09/2014		26/09/2008	05/08/2009		0	0	227	05/09/2014	0,9	695	21/02/2015	0,9	02/09/2015	0,9	02/09/2015	0,9	878	362								17/02/2016	08/07/2013				0	0	0	0	0	0	0	0	0	0	0	1	0	0	0	0	1	0	0	0	1	1	1	0	0	0
1	7771	18/11/2014	1	18/11/2014	EFV		07/01/2008		0	0	585	18/11/2014	0,9	897	21/05/2015	0,9	18/11/2015	0,9	18/11/2015	0,9	998	365								17/02/2016	07/01/2008				1	1	0	0	0	0	0	0	1	0	0	0	0	0	0	0	0	0	0	0	0	1	1	0	0	0
1	7878	19/11/2014	0	29/09/2014		01/01/2005	20/12/2008		0	0	147	29/09/2014	0,9	738	13/07/2015	0,9	08/01/2016	0,9	08/01/2016	0,9	940	466				1				17/02/2016	20/12/2008	06/02/2016 00:00		TOXY_NERV	0	0	0	0	0	0	0	0	1	0	0	1	0	0	0	0	0	0	0	0	1	1	1	0	0	0
1	7913	26/05/2014	1	08/10/2014	3TC	02/12/2008	16/12/2010		0	0	36	26/05/2014	39	142	05/12/2014	0,9	06/06/2015	39	09/12/2015	0,9	140	213,5								17/02/2016	14/01/2011				1	0	0	0	0	0	1	0	0	0	0	0	0	0	0	1	0	1	0	0	0	1	0	1	1	0
1	7928	19/09/2014	1	25/11/2014	3TC	06/06/1989	15/06/1990		1	0	449	19/09/2014	0,9	752	03/03/2015	0,9	02/09/2015	0,9	02/09/2015	0,9	586	281				1				17/02/2016	12/06/2006	21/09/2015 00:00	NO	SEMPLIFICAZIONE	1	0	0	1	0	0	0	0	0	0	0	0	0	0	0	0	0	1	0	0	1	1	0	1	0	0
1	7938	27/08/2014	1	31/10/2014	3TC	04/06/2008	18/11/2010		0	0	365	27/08/2014	0,9	1184	18/02/2015	0,9	20/08/2015	0,9	10/02/2016	93	660	380					10/02/2016 00:00	93	1	17/02/2016	15/05/2012				1	0	0	1	0	0	0	0	0	0	0	0	0	0	0	0	0	1	0	0	0	1	0	1	0	0
1	7941	23/10/2014	0	03/09/2014			20/04/2010		0	0	366	03/09/2014	0,9	939	02/03/2015	0,9			02/03/2015	0,9	686	180								17/02/2016	20/04/2010				0	0	0	0	0	0	0	0	1	0	0	1	0	0	0	0	0	0	0	0	1	1	1	0	0	0
1	7953	10/11/2014	0	12/09/2014		10/03/2009	04/07/2011		0	0	82	12/09/2014	0,9	726	03/02/2015	39	11/09/2015	0,9	11/09/2015	0,9	864	182								17/02/2016	25/11/2013				0	0	0	0	0	0	0	0	0	0	0	1	0	0	0	0	1	0	0	0	1	1	1	0	0	0
1	7954	09/09/2014	1	29/12/2014	EFV		14/09/2011		0	0	295	09/09/2014	0,9	693	16/02/2015	0,9	08/07/2015	0,9	03/11/2015	0,9	800	309								17/02/2016	14/09/2011				0	0	0	0	0	0	0	0	1	0	0	1	0	0	0	0	0	0	0	0	1	1	1	0	0	0
1	8027	29/09/2014	0	09/06/2014			07/03/2009		0	0	169	09/06/2014	0,9	539	01/12/2014	0,9	01/06/2015	0,9	01/12/2015	0,9	596	540								17/02/2016	27/05/2009				0	0	0	0	0	0	0	0	1	0	0	1	0	0	0	0	0	0	0	0	1	1	1	0	0	0
1	8058	08/10/2014	0	18/08/2014		18/05/2009	10/07/2009		0	1	279	18/08/2014	0,9	684	29/11/2014	0,9	24/04/2015	0,9	08/01/2016	0,9	777	508								17/02/2016	03/08/2010				0	0	0	0	0	0	0	0	0	1	0	1	0	0	0	0	0	0	0	0	1	1	1	0	0	0
1	8066	24/04/2014	1	06/10/2014	3TC	30/03/2009	22/12/2009		0	0	101	24/04/2014	0,9	302	13/11/2014	0,9	27/04/2015	39	08/10/2015	0,9	619	202,5								17/02/2016	30/01/2014				1	0	0	0	0	0	1	0	0	0	0	0	0	0	0	0	0	1	0	0	0	1	0	1	0	0
1	8087	07/11/2014	0	16/04/2014		01/01/2003	13/03/2003		0	0	155	16/04/2014	39	243	12/11/2014	39	04/02/2015	50	04/02/2015	50	280	0				1	04/02/2015 00:00	50	1	17/02/2016	18/03/2014	24/04/2015 00:00		SEMPLIFICAZIONE	0	0	0	0	0	0	0	0	0	1	0	1	0	0	0	1	0	0	0	0	1	1	1	0	1	0
9	8088	03/12/2014	0	15/04/2014		01/01/1995	01/10/1996	15/04/2014 00:00	0	0	345	15/04/2014	39	438	20/04/2015	39	23/10/2015	39	23/10/2015	39	506	0								17/02/2016	24/08/2009				0	0	0	0	0	0	0	0	0	0	0	1	0	0	1	0	0	0	0	0	1	1	1	0	0	0
1	8092	08/08/2014	1	12/11/2014	3TC	23/06/2009	15/06/2011		0	0	238	08/08/2014	0,9	464	14/01/2015	0,9	20/06/2015	0,9	06/02/2016	0,9	457	451								17/02/2016	27/06/2014				1	0	0	0	0	0	1	0	0	0	0	0	0	0	0	0	0	1	0	0	0	1	0	1	0	0
1	8121	31/10/2014	0	28/03/2014		01/11/2007	24/04/2009		9	0	366	28/03/2014	0,9	766	29/10/2014	0,9	14/05/2015	0,9	03/12/2015	0,9	794	615								17/02/2016	24/04/2009				0	0	0	0	0	0	0	0	1	0	0	1	0	0	0	0	0	0	0	0	1	1	1	0	0	0
1	8151	03/12/2014	0	22/09/2014		09/07/2009	07/09/2009	09/07/2009 00:00	0	0	26	22/09/2014	0,9	413	15/12/2014	0,9	09/03/2015	0,9	08/02/2016	0,9	421	504								17/02/2016	27/07/2011				1	1	0	1	0	0	0	0	0	0	0	0	0	0	0	0	0	1	0	0	0	1	0	1	0	0
1	8165	23/12/2014	0	02/09/2014		16/05/2009	07/09/2009		0	0	185	02/09/2014	39	678	05/12/2014	0,9	06/02/2015	0,9	15/01/2016	49	614	151,5								17/02/2016	01/10/2009				0	0	0	1	0	0	0	0	0	0	0	1	0	0	0	0	0	1	0	0	1	1	0	1	0	0
1	8173	03/11/2014	0	25/07/2014		01/01/1985	01/01/1993	14/02/1993 00:00	9	0	134	25/07/2014	0,9	385	11/02/2015	0,9	09/07/2015	0,9	09/07/2015	0,9	402	572								17/02/2016	26/03/2012				0	0	0	0	0	0	1	0	0	1	0	0	0	0	0	0	0	1	0	0	0	0	1	1	0	0
1	8210	01/12/2014	1	09/02/2015	3TC	19/10/2004	07/10/2009		0	0	495	01/12/2014	0,9	1539	21/05/2015	57	10/09/2015	0,9	10/09/2015	0,9	1268	106,5					21/05/2015 00:00	57	1	17/02/2016	14/04/2014				1	0	0	0	0	0	1	0	0	0	0	0	0	0	0	0	0	1	0	0	0	1	0	1	0	0
1	8240	24/02/2015	0	19/06/2014		17/09/2009	16/11/2011		0	0	394	19/06/2014	0,9	574	26/11/2014	0,9	27/05/2015	0,9	21/12/2015	0,9	768	550				1				17/02/2016	16/04/2012	13/01/2016 00:00		Co morbidity	0	0	0	0	0	0	1	0	0	0	0	1	0	0	0	0	0	1	0	0	0	1	0	1	0	0
1	8250	21/10/2014	0	09/07/2014		01/01/2000	24/11/2004	15/03/2005 00:00	0	0	384	09/07/2014	39	1169	13/12/2014	0,9	04/08/2015	0,9	03/12/2015	0,9	1188	433,5				1				17/02/2016	04/05/2010	22/12/2015 00:00		DISLIPIDEMIA	0	0	0	0	0	0	0	0	1	0	0	1	0	0	0	0	0	0	0	0	1	1	1	0	0	0
1	8289	10/10/2014	1	10/11/2014	3TC_AZT	28/09/2009	01/10/2009	15/10/2009 00:00	0	0	84	10/10/2014	39	722	09/03/2015	39			09/03/2015	39	461	0				1				17/02/2016	31/10/2014	30/03/2015 00:00	NO	SEMPLIFICAZIONE	1	0	0	0	1	0	1	0	0	0	0	0	0	0	0	1	0	1	0	0	0	1	0	1	1	0
9	8316	18/12/2014	0	04/09/2014			30/06/1998		0	0	475	04/09/2014	0,9	816	30/11/2015	39			30/11/2015	39	751	226								17/02/2016	15/12/2011				0	0	0	0	0	0	1	0	0	0	0	0	0	0	0	1	0	1	0	0	0	0	0	1	1	0
1	8322	26/09/2014	0	06/05/2014		01/12/2009	10/02/2010		0	0	249	06/05/2014	0,9	727								0				1				17/02/2016	10/02/2012	22/10/2014 00:00		SEMPLIFICAZIONE	0	0	0	0	0	0	0	0	0	0	0	1	1	0	0	0	0	0	0	0	1	1	0	1	0	0
1	8346	05/11/2014	0	22/09/2014		21/12/2009	19/03/2010		0	0	34	22/09/2014	39	323	09/03/2015	39	11/09/2015	0,9	11/09/2015	0,9	389	93								17/02/2016	07/06/2013				0	0	0	0	0	0	0	0	0	0	0	1	0	0	0	0	1	0	0	0	1	1	1	0	0	0
1	8355	14/10/2014	0	30/09/2014		19/12/2009	17/03/2010		0	0	260	30/09/2014	0,9	1015	10/12/2014	0,9	04/03/2015	0,9	03/06/2015	0,9	959	246				1				17/02/2016	17/03/2010	03/06/2015 00:00		SEMPLIFICAZIONE	0	0	0	1	0	0	0	0	0	0	0	1	0	0	0	0	0	1	0	0	1	1	0	1	0	0
1	8391	23/09/2014	1	03/12/2014	3TC	04/02/2010	25/03/2010		0	0	17	23/09/2014	0,9	632	25/03/2015	0,9	17/04/2015	0,9	17/04/2015	0,9	828	135				1				17/02/2016	11/10/2010	19/05/2015 00:00	3TC	ESCAPE	1	0	0	1	0	0	0	0	0	0	0	0	0	0	0	0	0	0	0	0	0	1	0	1	0	0
1	8409	07/11/2014	0	30/04/2014		05/02/2007	15/02/2007		0	0	368	30/04/2014	0,9	740	18/10/2014	0,9	28/03/2015	0,9	29/08/2015	0,9	1013	486								17/02/2016	06/11/2009				1	1	0	1	0	0	0	0	0	0	0	0	0	0	0	0	0	1	0	0	0	1	0	1	0	0
1	8431	08/08/2014	1	03/11/2014	3TC	30/08/1996	30/08/1996		1	0	329	08/08/2014	0,9	712	24/12/2014	0,9	03/03/2015	0,9	03/03/2015	0,9	852	120				1				17/02/2016	31/10/2013	30/04/2015 00:00	NO	PATIENT	1	0	0	1	0	0	0	0	0	0	0	0	0	0	0	0	0	1	0	0	0	1	0	1	0	0
2	8471	21/08/2014	1	14/11/2014	3TC	26/05/2010	12/07/2010		0	0	199	21/08/2014	39	766	14/02/2015	39	20/06/2015	39	21/11/2015	0,9	813	77				1				17/02/2016	13/07/2010	01/12/2015 00:00	NO	SEMPLIFICAZIONE	1	0	0	0	0	0	1	0	0	0	0	0	0	0	0	1	0	1	0	0	0	1	0	1	1	0
1	8475	07/10/2014	0	08/08/2014			08/07/2010		0	0	154	08/08/2014	39	405	31/10/2014	57	17/11/2014	39	02/01/2016	0,9	374	218				1	31/10/2014 00:00	57	1	17/02/2016	20/07/2012	11/01/2016 00:00		DISLIPIDEMIA	0	0	0	1	0	0	0	0	0	0	0	1	0	0	0	0	0	1	0	0	1	1	0	1	0	0
1	8497	02/10/2014	0	29/04/2014		10/05/2006	09/09/2010		0	0	304	29/04/2014	39	792	09/10/2014	39	11/04/2015	0,9	21/10/2015	0,9	808	285								17/02/2016	11/01/2012				0	0	0	1	0	0	0	0	0	0	0	1	0	0	0	0	0	1	0	0	0	1	0	1	0	0
1	8518	09/02/2015	0	12/05/2014		01/10/1995	27/05/2010		0	0	504	12/05/2014	0,9	1548	26/02/2015	0,9	21/07/2015	0,9	15/01/2016	0,9	1561	613								17/02/2016	07/09/2012				0	0	0	1	0	0	0	0	0	0	0	1	0	0	0	0	0	1	0	0	1	1	0	1	0	0
1	8519	12/11/2014	0	24/09/2014		11/06/2010	09/08/2010		0	0	99	24/09/2014	0,9	545	17/12/2014	0,9			17/12/2014	0,9	908	84				1				17/02/2016	02/08/2013	17/12/2014 00:00		DISLIPIDEMIA	1	0	0	1	0	0	0	0	0	0	0	0	0	0	0	0	0	1	0	0	0	1	0	1	0	0
1	8520	02/04/2014	1	28/10/2014	3TC	30/06/1985	01/01/1985		1	9	195	02/04/2014	39	387	25/11/2014	0,9	05/06/2015	0,9	29/01/2016	0,9	694	444								17/02/2016	14/05/2014				1	0	0	1	0	0	0	0	0	0	0	0	0	0	0	0	0	1	0	0	0	1	0	1	0	0
1	8530	21/03/2014	1	04/11/2014	3TC	01/01/1997	01/02/2002		0	0	931	21/03/2014	39	1191	28/01/2015	0,9	04/09/2015	0,9	04/09/2015	0,9	1207	261,5				1				17/02/2016	15/03/2012	10/09/2015 00:00	NO	TOXY_LIVER	1	0	0	1	0	0	0	0	0	0	0	0	0	0	0	0	0	1	0	0	0	1	0	1	0	0
9	8544	24/11/2014	0	04/08/2014		01/01/1998	31/12/1999		0	0	424	04/08/2014	0,9	582	22/01/2015	0,9	07/07/2015	0,9	07/07/2015	0,9	597	337								17/02/2016	13/02/2014				0	0	0	0	0	0	0	0	0	0	0	1	0	0	0	0	1	0	0	0	1	1	1	0	0	0
1	8548	22/08/2014	1	19/12/2014	EFV	15/06/1992	28/11/1992		9	0	467	22/08/2014	39	702	16/01/2015	0,9	12/06/2015	0,9	20/11/2015	39	689	241,5								17/02/2016	02/09/2010				1	1	0	0	0	0	0	0	1	0	0	0	0	0	0	1	0	0	0	0	0	1	1	0	1	0
1	8549	15/07/2014	1	28/10/2014	3TC	05/08/2010	04/08/2011		0	0	314	15/07/2014	47	750	13/11/2014	0,9	01/04/2015	0,9	12/11/2015	0,9	544	372								17/02/2016	18/04/2014				1	0	0	0	0	0	1	0	0	0	0	0	0	0	0	0	0	1	0	0	0	1	0	1	0	0
1	8555	30/05/2014	1	07/11/2014	3TC	24/09/2010	30/09/2010		0	0	381	30/05/2014	0,9	794	18/03/2015	0,9	03/11/2015	0,9	03/11/2015	0,9	964	361				1				17/02/2016	31/08/2012	15/12/2015 00:00	ALL	SEMPLIFICAZIONE	1	0	0	0	0	0	1	0	0	0	0	0	0	0	0	0	0	1	0	0	0	1	0	1	0	0
1	8559	31/10/2014	0	08/08/2014		20/08/2010	17/04/2012		0	0	416	08/08/2014	49	1171	31/10/2014	49	23/01/2015	49	23/01/2015	49	784	0								17/02/2016	17/04/2012				0	0	0	0	0	0	0	1	0	0	0	1	0	0	0	0	0	0	0	0	1	1	0	0	0	0
1	8567	20/10/2014	0	17/05/2014		15/03/1998	15/03/1998		0	0	1244	17/05/2014	0,9	1502	12/11/2014	0,9	08/05/2015	0,9	14/11/2015	0,9	2102	546								17/02/2016	16/12/2013				0	0	0	0	0	0	0	0	0	0	0	1	0	0	0	0	1	0	0	0	1	1	1	0	0	0
1	8617	25/01/2014	1	02/12/2014	EFV		09/11/2010		0	0	515	25/01/2014	36	708	22/01/2015	0,9	03/07/2015	61	25/01/2016	0,9	638	209,5					03/07/2015 00:00	61	1	17/02/2016	09/11/2010				0	0	0	0	0	0	0	1	0	0	0	1	0	0	0	0	0	0	0	0	1	1	1	0	0	0
1	8623	07/11/2014	1	25/11/2014	3TC_AZT	01/11/2010	23/12/2010	01/03/2011 00:00	0	0	34	07/11/2014	0,9	159	02/02/2015	0,9	27/05/2015	54	02/10/2015	0,9	182	204,5					27/05/2015 00:00	54	1	17/02/2016	02/05/2011				1	0	0	0	1	0	1	0	0	0	0	0	0	1	0	0	0	1	0	0	0	1	0	1	0	1
1	8625	19/11/2014	0	05/08/2014		01/01/2001	30/06/2001		1	0	923	05/08/2014	0,9	1337	29/01/2015	0,9	16/10/2015	0,9	16/10/2015	0,9	722	437				1				17/02/2016	15/07/2007	29/10/2015 00:00		MORTE	0	0	0	0	0	0	0	0	0	0	1	1	0	0	0	0	0	1	0	0	1	1	0	1	0	0
1	8629	03/10/2014	0	06/05/2014		25/10/2010	23/12/2010		0	0	201	06/05/2014	39	621	19/11/2014	51	06/03/2015	39	14/09/2015	39	633	0					19/11/2014 00:00	51	1	17/02/2016	16/12/2013				0	0	0	0	0	0	0	0	0	0	0	1	0	0	0	0	1	0	0	0	1	1	1	0	0	0
1	8639	30/10/2014	0	22/08/2014		25/10/2010	13/05/2013		0	9	288	22/08/2014	0,9	678	17/12/2014	39	16/06/2015	0,9	22/12/2015	0,9	948	338								17/02/2016	13/05/2013				0	0	0	0	0	0	0	0	0	0	0	1	0	0	0	0	1	0	0	0	1	1	1	0	0	0
1	8652	12/08/2014	1	30/01/2015	3TC		01/01/1994		1	0	397	12/08/2014	0,9	456	07/07/2015	0,9	01/02/2016	0,9	01/02/2016	0,9	316	367								17/02/2016	01/09/2011				1	0	0	1	0	0	0	0	0	0	0	0	0	0	0	0	0	1	0	0	0	1	0	1	0	0
1	8684	08/10/2014	1	08/10/2014	3TC		17/06/2011		0	0	293	08/10/2014	0,9	665	05/05/2015	0,9	20/10/2015	0,9	20/10/2015	0,9	871	377								17/02/2016	10/02/2014				1	0	0	1	0	0	0	0	0	0	0	0	0	0	0	0	0	1	0	0	0	1	0	1	0	0
1	8697	27/10/2014	1	27/10/2014	3TC	22/12/2010	08/11/2012		0	0	478	27/10/2014	0,9	782	08/04/2015	0,9	10/09/2015	0,9	11/01/2016	0,9	757	441								17/02/2016	15/04/2014				1	0	0	0	0	0	1	0	0	0	0	0	0	0	0	0	0	1	0	0	0	1	0	1	0	0
1	8735	12/11/2014	0	26/03/2014		21/01/2011	26/05/2011		0	0	243	26/03/2014	0,9	447	09/10/2014	0,9	04/04/2015	0,9	04/12/2015	61	566	464,5	02/10/2015 00:00	104	1	1				17/02/2016	27/05/2013	04/12/2015 00:00		VIRAL_FAILURE	0	0	0	0	0	0	0	0	0	0	0	1	0	0	0	0	1	0	0	0	1	1	1	0	0	0
1	8765	17/09/2014	1	14/10/2014	3TC_AZT	01/01/1988	01/01/2006		1	0	1011	17/09/2014	0,9	1019	20/11/2014	0,9			20/11/2014	0,9	1207	37				1				17/02/2016	11/12/2013	27/11/2014 00:00	COMBI	TOXY_LIVER	1	0	0	0	1	0	0	0	0	0	0	0	0	0	0	1	0	0	0	0	0	1	0	0	1	0
1	8771	29/08/2014	1	17/11/2014	EFV	21/04/1999	03/05/2011		9	9	711	29/08/2014	0,9	720								0				1				17/02/2016	03/05/2011	26/01/2015 00:00	ALL	HYPERSENSITIVITY	0	0	0	0	0	0	0	0	1	0	0	0	0	0	0	1	0	0	0	0	1	1	1	0	1	0
1	8778	14/10/2014	0	08/09/2014		01/01/1990	25/02/2008		1	0	843	08/09/2014	39	937	29/01/2015	77	09/03/2015	0,9	04/11/2015	39	1266	196					29/01/2015 00:00	77	1	17/02/2016	16/06/2011				0	0	0	1	0	0	0	0	0	0	0	0	0	1	0	1	0	1	0	0	0	0	0	1	1	1
1	8793	13/10/2014	1	24/10/2014	3TC	29/03/2011	12/07/2011		0	0	5	13/10/2014	0,9	563	16/02/2015	0,9	08/06/2015	0,9	30/12/2015	0,9	510	432								17/02/2016	12/07/2011				1	0	0	0	0	0	1	0	0	0	0	0	0	0	0	1	0	1	0	0	0	1	0	1	1	0
1	8794	09/10/2014	0	31/05/2014		09/02/2011	06/07/2011		0	0	232	31/05/2014	0,9	646	08/11/2014	0,9	04/05/2015	0,9	30/10/2015	0,9	733	517								17/02/2016	11/12/2013				0	0	0	0	0	0	0	0	0	0	0	1	0	0	0	0	1	0	0	0	1	1	1	0	0	0
1	8844	05/12/2014	0	25/07/2014			15/06/1995		9	9	82	25/07/2014	49	442	17/10/2014	49	09/01/2015	49	09/01/2015	49	507	0								17/02/2016	09/12/2011				0	1	0	0	0	0	1	1	0	1	0	0	0	0	0	0	0	1	0	0	0	1	1	1	0	0
1	8864	30/06/2014	1	07/10/2014	3TC		01/07/2011	15/07/2011 00:00	0	9	159	30/06/2014	0,9	449	16/10/2014	0,9	14/02/2015	0,9	13/02/2016	0,9	498	494								17/02/2016	19/08/2014				1	0	0	0	0	0	1	0	0	0	0	0	0	0	0	0	0	1	0	0	0	1	0	1	0	0
9	8867	14/11/2014	0	11/06/2014		24/05/2011	22/08/2011		0	0	258	11/06/2014	0,9	750	24/11/2014	0,9			24/11/2014	0,9	868	166								17/02/2016	15/06/2012	20/01/2015 00:00		SEMPLIFICAZIONE	0	0	0	0	0	0	0	0	0	0	0	1	0	0	0	1	0	0	0	0	1	1	0	0	1	0
1	8899	31/10/2014	0	20/07/2014			01/01/1992	02/08/2011 00:00	9	9	19	20/07/2014	19	320	20/10/2014	19			20/10/2014	19	396	0								17/02/2016	24/05/2012				0	0	0	0	0	0	1	0	0	1	0	0	0	1	0	0	0	1	0	1	0	0	1	1	0	1
1	8925	03/12/2014	0	24/04/2014		05/05/2001	29/04/2013		0	0	424	24/04/2014	0,9	614	15/10/2014	39	03/03/2015	287	11/01/2016	72		87				1	03/03/2015 00:00	287	1	17/02/2016	29/04/2013	18/01/2016 00:00		OTHER	0	0	0	0	0	0	0	0	0	0	0	1	0	0	0	0	1	0	0	0	1	1	1	0	0	0
1	8932	08/10/2014	0	11/06/2014		27/09/2011	10/11/2011		0	0	22	11/06/2014	39	142	11/11/2014	39			11/11/2014	39	135	0				1				17/02/2016	10/11/2011	28/11/2014 00:00		IMMUNOLOGICAL	0	0	0	0	0	0	1	0	0	0	0	1	0	0	0	0	0	1	0	0	1	1	0	1	0	0
1	8935	17/11/2014	0	13/05/2014		01/04/2006	01/09/2007		9	9	584	13/05/2014	0,9	707	15/10/2014	0,9	14/03/2015	0,9	19/09/2015	0,9	800	494								17/02/2016	01/09/2007				0	0	0	0	0	0	0	0	0	0	0	1	0	0	1	0	0	0	0	0	1	1	1	0	0	0
1	8949	14/11/2014	1	14/11/2014	3TC_AZT	10/08/2004	15/08/2004	10/08/2004 00:00	0	0	518	14/11/2014	0,9	967	05/02/2015	0,9	10/06/2015	0,9	02/12/2015	0,9	1592	383								17/02/2016	11/01/2013				1	0	0	0	1	0	0	1	0	0	0	0	0	1	0	0	0	0	0	0	0	1	0	0	0	1
1	8966	13/08/2014	1	30/10/2014	3TC	08/11/2011	28/12/2011		0	0	276	13/08/2014	0,9	680	05/01/2015	0,9	10/03/2015	0,9	11/01/2016	0,9	711	438								17/02/2016	04/04/2014				1	0	0	0	0	0	1	0	0	0	0	0	0	0	0	0	0	1	0	0	0	1	0	1	0	0
1	8976	29/10/2014	0	03/09/2014		01/01/1995	01/11/1995		0	0	483	03/09/2014	39	706	03/03/2015	0,9	17/09/2015	0,9	17/09/2015	0,9	729	288,5								17/02/2016	08/02/2012				0	0	0	1	0	0	0	0	0	0	0	0	0	0	1	0	0	1	0	0	0	0	1	1	0	0
1	9035	03/07/2014	1	17/11/2014	3TC_AZT	22/02/2012	30/03/2012		9	9	325	03/07/2014	0,9	482	21/11/2014	0,9	27/03/2015	0,9	14/10/2015	0,9	667	331								17/02/2016	10/04/2012				1	0	0	0	1	0	0	0	0	0	0	0	0	0	0	1	0	0	0	0	0	1	0	0	1	0
1	9039	13/11/2014	0	19/06/2014		02/02/2012	05/02/2012	17/02/2012 00:00	0	0	88	19/06/2014	0,9	554	30/12/2014	0,9			30/12/2014	0,9	605	194				1				17/02/2016	21/09/2012	15/01/2015 00:00		SEMPLIFICAZIONE	0	0	0	0	0	0	0	1	0	0	0	1	0	0	0	1	0	0	0	0	1	1	0	0	1	0
1	9047	03/09/2014	1	19/11/2014	EFV	01/02/2008	01/03/2008		0	9	829	03/09/2014	0,9	969	18/04/2015	39	06/11/2015	0,9	06/11/2015	0,9	1162	176								17/02/2016	01/03/2008				0	0	0	0	0	0	0	0	1	0	0	1	0	0	0	0	0	0	0	0	1	1	1	0	0	0
2	9059	03/06/2014	1	14/10/2014	3TC_AZT	01/01/2002	01/02/2004		0	0	670	03/06/2014	0,9	1709	19/11/2014	0,9	26/05/2015	0,9	27/10/2015	0,9	1310	378								17/02/2016	31/07/2013				1	0	0	0	1	0	0	0	0	0	0	0	0	0	1	0	0	0	0	0	0	1	1	0	0	0
1	9071	28/11/2014	0	09/04/2014		15/11/1996	06/06/2005		1	0	612	09/04/2014	39	658	09/10/2014	64			09/10/2014	64	746	0				1	09/10/2014 00:00	64	1	17/02/2016	10/02/2014	20/10/2014 00:00		SEMPLIFICAZIONE	0	0	0	0	0	0	1	0	0	0	0	1	0	0	0	0	0	1	0	0	1	1	0	1	0	0
1	9105	17/11/2014	0	28/07/2014		01/01/2012	24/08/2012		9	9	250	28/07/2014	0,9	626	25/02/2015	39	08/07/2015	0,9	22/12/2015	0,9	712	339,5				1				17/02/2016	09/08/2013	28/12/2015 00:00		TOXY_OSTEO	0	0	0	1	0	0	0	0	0	0	0	1	0	0	0	0	0	1	0	0	1	1	0	1	0	0
1	9384	01/08/2014	1	28/11/2014	EFV	02/10/2006	30/09/2009		1	0	623	01/08/2014	0,9	623	20/01/2015	0,9	31/07/2015	0,9	31/07/2015	0,9	1091	245								17/02/2016	20/12/2013				1	1	0	0	0	0	0	0	1	0	0	0	0	0	0	0	0	0	0	0	0	1	1	0	0	0
1	9395	24/10/2014	0	18/06/2014		02/10/2002	15/10/2002		0	0	695	18/06/2014	0,9	1013	20/11/2014	0,9	12/05/2015	0,9	21/01/2016	0,9	871	582								17/02/2016	02/01/2014				1	1	0	0	0	0	0	0	0	0	0	0	0	0	0	0	1	0	0	0	0	1	1	0	0	0
3	9428	05/11/2014	0	08/04/2014		01/01/2000	01/01/1998	01/01/2000 00:00	9	9	624	08/04/2014	0,9	704	11/10/2014	0,9	21/03/2015	0,9	14/10/2015	0,9	773	554								17/02/2016	01/01/2010				0	0	0	0	0	0	0	0	1	0	0	1	0	0	0	0	0	0	0	0	1	1	1	0	0	0
1	9469	19/11/2014	1	19/11/2014	3TC	01/01/1989	01/01/1999		1	0	1178	19/11/2014	0,9	1747	16/04/2015	0,9			16/04/2015	0,9	1742	148								17/02/2016	30/04/2014				1	0	0	0	0	0	0	0	0	0	0	0	1	0	0	0	0	0	0	0	0	1	0	1	0	0
9	9484	14/10/2014	0	11/10/2014		01/01/1985	01/01/1992		1	0	388	11/10/2014	0,9	649	14/03/2015	0,9	19/09/2015	0,9	19/09/2015	0,9	818	343								17/02/2016	31/12/2008				0	0	0	1	0	0	0	0	0	0	0	0	0	0	0	1	0	0	0	0	0	0	0	1	1	0
1	9495	07/11/2014	0	28/08/2014		30/06/1991	15/03/2004	15/03/2003 00:00	9	9	574	28/08/2014	39	906	07/03/2015	0,9	26/08/2015	0,9	09/02/2016	0,9	1517	434,5								17/02/2016	13/04/2010				0	0	0	0	0	0	1	0	0	0	0	1	0	0	0	1	0	1	0	0	1	1	0	1	1	0
1	9497	02/12/2014	0	23/09/2014		13/09/2012	30/04/2014		0	0	489	23/09/2014	0,9	711	09/02/2015	0,9	28/08/2015	0,9	28/08/2015	0,9	1008	425								17/02/2016	30/04/2014				0	0	0	0	0	0	0	0	0	0	0	1	0	0	0	0	1	0	0	0	1	1	1	0	0	0
1	9504	17/11/2014	0	09/09/2014		09/03/1980	01/01/2001		0	9	416	09/09/2014	39	539	10/03/2015	0,9	14/09/2015	0,9	14/09/2015	0,9	647	279								17/02/2016	01/01/2010				0	0	0	0	0	0	0	0	0	1	0	0	0	0	0	1	0	0	0	0	0	0	1	0	1	0
2	9514	25/08/2014	1	25/11/2014	3TC	02/10/2012	21/12/2012		0	0	389	25/08/2014	0,9	389	05/01/2015	0,9	07/07/2015	0,9	21/12/2015	0,9	646	391								17/02/2016	03/04/2014				1	0	0	0	0	0	1	0	0	0	0	0	0	0	0	0	0	1	0	0	0	1	0	1	0	0
1	9519	31/10/2014	0	23/04/2014		15/11/1997	15/11/1997		1	0	814	23/04/2014	33	1267	16/10/2014	144	13/11/2014	0,9	17/08/2015	19	1572	152,5					16/10/2014 00:00	144	1	17/02/2016	11/10/2012				1	1	0	1	0	0	0	0	0	0	0	0	0	0	0	0	0	1	0	0	0	1	0	1	0	0
1	9525	05/07/2014	1	09/10/2014	3TC	01/01/1992	13/12/2012		1	0	290	05/07/2014	0,9	403	21/01/2015	39	11/07/2015	46	08/01/2016	40	476	52								17/02/2016	10/02/2014				1	0	0	1	0	0	0	0	0	0	0	0	0	0	0	0	0	1	0	0	0	1	0	1	0	0
9	9542	19/11/2014	0	28/10/2014		01/12/1984	01/06/1996		1	0	545	28/10/2014	0,9	713	04/06/2015	0,9	27/10/2015	0,9	27/10/2015	0,9	623	364								17/02/2016	15/06/2003				1	1	0	1	0	0	0	0	0	0	0	0	0	0	0	0	0	1	0	0	0	1	0	1	0	0
1	9548	08/10/2014	0	06/05/2014		08/11/2012	14/03/2013		0	0	297	06/05/2014	0,9	392	03/10/2014	0,9			03/10/2014	0,9	457	150				1				17/02/2016	14/03/2013	12/12/2014 00:00		PATIENT	0	0	0	0	0	0	0	0	0	0	0	1	0	0	0	0	1	0	0	0	1	1	1	0	0	0
1	9567	30/09/2014	0	20/06/2014		15/06/2000	15/06/2004		0	0	496	20/06/2014	0,9	608								0				1				17/02/2016	12/02/2012	20/08/2014 00:00		SEMPLIFICAZIONE	0	0	0	0	0	0	0	0	0	0	0	1	0	0	1	0	0	0	0	0	1	1	1	0	0	0
1	9582	04/11/2014	0	12/05/2014		01/06/1998	01/06/1998		0	9	928	12/05/2014	0,9	1040	14/10/2014	0,9	02/01/2015	39	31/10/2015	0,9	1085	458,5								17/02/2016	09/05/2013				0	0	0	0	0	0	0	0	0	0	0	1	1	0	0	0	0	0	0	0	1	1	0	1	0	0
1	9585	03/11/2014	0	26/06/2014		01/01/1993	14/01/2003		1	0	284	26/06/2014	0,9	786	09/10/2014	0,9	19/03/2015	44	02/12/2015	0,9	576	411,5								17/02/2016	19/02/2013				0	0	0	0	0	0	1	0	0	0	0	1	0	0	0	0	0	1	0	0	1	1	0	1	0	0
1	9592	07/10/2014	0	27/09/2014		29/09/1994	01/10/1996		1	0	122	27/09/2014	0,9	333	28/02/2015	0,9	29/05/2015	0,9	02/12/2015	0,9	466	431								17/02/2016	01/12/2012				0	0	0	0	0	0	1	0	0	0	0	1	0	0	0	0	0	1	0	0	1	1	0	1	0	0
9	9615	29/10/2014	0	28/08/2014			01/01/1992		9	9	110	28/08/2014	49	322								0								17/02/2016	11/06/2013				0	0	0	0	0	0	0	0	0	1	0	0	0	1	0	0	0	0	0	0	0	0	1	0	0	1
1	9620	14/11/2014	0	22/09/2014		18/04/2013	03/12/2013		0	0	306	22/09/2014	0,9	510	12/03/2015	0,9	21/09/2015	0,9	21/09/2015	0,9	597	364								17/02/2016	03/12/2013				0	0	0	0	0	0	0	0	0	0	0	1	0	0	0	0	1	0	0	0	1	1	1	0	0	0
1	9632	27/10/2014	0	29/05/2014		24/04/2013	24/05/2013		0	0	466	29/05/2014	0,9	779	31/10/2014	0,9	15/05/2015	0,9	23/12/2015	0,9	798	573								17/02/2016	15/10/2013				0	0	0	0	0	0	0	0	0	0	0	1	0	0	0	1	0	0	0	0	1	1	0	0	1	0
1	9657	04/09/2014	1	03/11/2014	3TC	01/01/1988	01/01/2002		1	0	490	04/09/2014	0,9	557	30/12/2014	0,9	14/05/2015	0,9	14/05/2015	0,9	552	192				1				17/02/2016	19/09/2014	27/05/2015 00:00	ALL	DRUG_INT	1	0	0	1	0	0	0	0	0	0	0	0	0	0	0	0	0	1	0	0	0	1	0	1	0	0
1	9664	30/01/2015	0	03/09/2014			30/06/1998		1	0	373	03/09/2014	0,9	466	18/03/2015	0,9	01/07/2015	0,9	07/10/2015	0,9	469	399								17/02/2016	14/03/2013				0	0	0	0	0	0	1	0	0	0	0	1	0	0	0	0	0	1	0	0	1	1	0	1	0	0
1	9682	04/11/2014	0	10/06/2014		01/06/2005	01/06/2008		0	0	662	10/06/2014	0,9	1005	12/12/2014	39	15/05/2015	39	09/10/2015	0,9	972	166								17/02/2016	13/02/2014				1	1	0	0	0	0	0	0	0	0	0	0	0	0	0	0	1	0	0	0	0	1	1	0	0	0
1	9683	19/11/2014	0	23/06/2014		01/01/1996	01/01/1996		1	0	398	23/06/2014	39	572	04/12/2014	0,9	05/05/2015	39	26/10/2015	39	515	158								17/02/2016	02/07/2010				0	0	0	1	0	0	0	0	0	0	0	1	0	0	0	0	0	0	0	0	1	1	0	1	0	0
9	9701	14/10/2014	0	22/05/2014		22/03/2013	27/11/2013		0	0	410	22/05/2014	0,9	600	21/11/2014	0,9	22/04/2015	0,9	27/10/2015	0,9	678	523								17/02/2016	27/11/2013				0	0	0	0	0	0	0	0	0	0	0	1	0	0	0	0	1	0	0	0	1	1	1	0	0	0
1	9708	01/08/2013	1	05/01/2015	EFV	01/01/2008	01/01/2008		0	0	745	01/08/2013	0,9	745	29/04/2015	2644	07/01/2016	39	07/01/2016	39	553	57					29/04/2015 00:00	2644	1	17/02/2016	01/01/2008				1	1	0	0	0	0	0	0	1	0	0	0	0	0	0	0	0	0	0	0	0	1	1	0	0	0
1	9754	20/11/2014	0	07/04/2014		01/02/2006	01/02/2006		9	0	477	07/04/2014	39	607	11/11/2014	39	22/05/2015	0,9	04/11/2015	0,9	520	262								17/02/2016	01/02/2006				0	0	0	0	0	0	0	0	1	0	0	0	0	0	0	0	0	0	0	0	1	1	1	0	0	0
1	9764	25/11/2014	0	29/08/2014		29/08/2013	03/10/2013	29/08/2013 00:00	0	0	24	29/08/2014	0,9	205	24/11/2014	0,9	16/02/2015	0,9	16/02/2015	0,9	381	171				1				17/02/2016	03/10/2013	16/02/2015 00:00		STUDY	0	0	0	0	0	0	0	0	0	0	0	1	0	1	0	1	0	0	0	0	1	1	0	0	1	1
2	10012	19/06/2014	1	19/11/2014	3TC	01/01/1995	01/01/1995		1	0	749	19/06/2014	39	749	16/03/2015	48			16/03/2015	48	810	0								17/02/2016	01/11/2012				1	0	0	0	0	0	1	0	0	0	0	0	0	0	0	0	0	1	0	0	0	1	0	1	0	0
1	10057	19/11/2014	0	29/08/2014		01/08/2006	01/03/2008		0	0	853	29/08/2014	39	866								0				1				17/02/2016	01/03/2009	15/09/2014 00:00		TOXY_RENALE	0	0	0	0	0	0	0	0	1	0	0	1	0	0	0	0	0	0	0	0	1	1	1	0	0	0
1	10061	05/01/2015	0	22/09/2014		19/09/2005	24/02/2009		0	9	612	22/09/2014	0,9	725								0				1				17/02/2016	15/05/2011	03/10/2014 00:00		SEMPLIFICAZIONE	0	0	0	1	0	0	0	0	0	0	0	1	0	0	0	0	0	1	0	0	1	1	0	1	0	0
1	10063	15/12/2014	0	18/09/2014		15/09/1986	15/06/2000		0	0	751	18/09/2014	0,9	776	16/02/2015	39	10/10/2015	0,9	10/10/2015	0,9	684	0				1				17/02/2016	15/03/2008	01/11/2015 00:00		SEMPLIFICAZIONE	0	0	0	0	0	0	0	0	0	0	0	1	0	0	1	0	0	0	0	0	1	1	1	0	0	0
